# The contribution of white matter pathology, hypoperfusion, lesion load, and stroke recurrence to language deficits following acute subcortical left hemisphere stroke

**DOI:** 10.1371/journal.pone.0275664

**Published:** 2022-10-26

**Authors:** Massoud S. Sharif, Emily B. Goldberg, Alexandra Walker, Argye E. Hillis, Erin L. Meier

**Affiliations:** 1 Department of Neurology, Johns Hopkins University, Baltimore, Maryland, United States of America; 2 Department of Physical Medicine and Rehabilitation, Johns Hopkins University, Baltimore, Maryland, United States of America; 3 Department of Cognitive Science, Johns Hopkins University, Baltimore, Maryland, United States of America; ICREA, University of Barcelona, SPAIN

## Abstract

Aphasia, the loss of language ability following damage to the brain, is among the most disabling and common consequences of stroke. Subcortical stroke, occurring in the basal ganglia, thalamus, and/or deep white matter can result in aphasia, often characterized by word fluency, motor speech output, or sentence generation impairments. The link between greater lesion volume and acute aphasia is well documented, but the independent contributions of lesion location, cortical hypoperfusion, prior stroke, and white matter degeneration (leukoaraiosis) remain unclear, particularly in subcortical aphasia. Thus, we aimed to disentangle the contributions of each factor on language impairments in left hemisphere acute subcortical stroke survivors. Eighty patients with acute ischemic left hemisphere subcortical stroke (less than 10 days post-onset) participated. We manually traced acute lesions on diffusion-weighted scans and prior lesions on T2-weighted scans. Leukoaraiosis was rated on T2-weighted scans using the Fazekas et al. (1987) scale. Fluid-attenuated inversion recovery (FLAIR) scans were evaluated for hyperintense vessels in each vascular territory, providing an indirect measure of hypoperfusion in lieu of perfusion-weighted imaging. We found that language performance was negatively correlated with acute/total lesion volumes and greater damage to substructures of the deep white matter and basal ganglia. We conducted a LASSO regression that included all variables for which we found significant univariate relationships to language performance, plus nuisance regressors. Only total lesion volume was a significant predictor of global language impairment severity. Further examination of three participants with severe language impairments suggests that their deficits result from impairment in domain-general, rather than linguistic, processes. Given the variability in language deficits and imaging markers associated with such deficits, it seems likely that subcortical aphasia is a heterogeneous clinical syndrome with distinct causes across individuals.

## Introduction

Aphasia, the loss of language ability following injury to the brain, is a common and often disabling consequence of acute left hemisphere (LH) cortical stroke [1–3]. Aphasia has been reported in frequencies as high as 62% in acute ischemic stroke—a higher rate than in hemorrhagic stroke or in ischemic stroke at any other time point [2].

The neural substrates of cortical aphasia are well-established [4,5]. Subcortical aphasia, which occurs after strokes in the basal ganglia, thalamus, or deep white matter, is not as well characterized [6–8]. Subcortical aphasia lacks some of the clinical features of classic aphasia subtypes [9], and the type and severity of language impairments after subcortical stroke varies widely [7,10]—with no characteristic error pattern found among these patients generally [11]. Whether subcortical structures have a direct role in processing language is controversial [12]. The basal ganglia may be involved in the formulation and selection of language segments or merely in the motoric aspects of their release [13]. Similarly, the thalamus may be crucial to integrative language functions or may only support the attentional mechanisms that underlie them [13]. Here, further historical context is appropriate.

Nearly a century and a half ago, Broadbent believed the cells of the striatum store pre-formed articulatory patterns for spoken words [14]. Others maintained that the basal ganglia mediated purely motoric functions [15], and the idea that subcortical aphasia was merely the result of disconnecting cortical language areas emerged as well [16]. In the mid-twentieth century, before the emergence of modern imaging, speech disturbances during intraoperative stimulation or ablation of the thalamus and basal ganglia in Parkinsonian patients continued this line of inquiry [17,18]. More recently, a study of language in Huntington’s disease patients suggested that the contributions of the striatum to syntax processing are not merely a reflection of its role in working memory [19]. In a recent review, however, Radanovic and Mansur [8] argue that neurodegenerative diseases such as Parkinson’s and Huntington’s, while both diseases of the basal ganglia, are imperfect lesion models for explaining the role of these structures in language processing. They explain that language impairments are better attributed to the diffuse effect of these pathologies on the cerebral cortex.

Evidence from subcortical stroke itself has also contributed to this debate. Kuljic-Obradovic [20], in a study of 32 aphasic patients with LH subcortical strokes, proposed a division of subcortical aphasia into three syndromes. He proposed patients with two types (i.e., striatocapsular aphasia and aphasia associated with periventricular white matter lesions) display similar patterns of impairment in speech fluency and shortened phrase length with preserved comprehension and naming. Individuals with thalamic aphasia, on the other hand, present with fluent output but impaired comprehension and naming. Kuljic-Obradovic [20] found that repetition is generally spared across these proposed subtypes. This dissociation, the author argues, suggests a phonetic processing role for the basal ganglia and a lexical-semantic processing role for the thalamus. In addition, Crosson et al. [21] found that the left basal ganglia may have a role in reorganizing language production faculties to the right hemisphere after ischemic LH stroke.

More recent work has provided evidence that subcortical structures only indirectly support language. Bohali & Crosson [22] discuss two major functional loops through the basal ganglia and thalamus that support lexical selection and articulation. The first of these loops connects the left pre-supplementary motor area, dorsal caudate, and ventral anterior thalamus, while the second connects Broca’s area to the basal ganglia. The authors implicate the first loop in the retrieval of words from pre-existing lexical stores. Here, the role of the basal ganglia is to refine contrasts between desired and non-desired words in order to increase the signal-to-noise ratio [22]. Similarly, the second loop refines the contrast between desired and competing phonological/articulatory representations. Together, the two loops ensure the accurate selection and proper articulation of the desired lexical phrases. This explanation accords with a recent review by Nadeau [23], who emphasized the strictly computational and non-data-specific functions of the basal ganglia in assisting language. The basal ganglia, Nadeau continues, do not have strict language functionalities, with the possible exception of representing movement verbs. Further, a review by Shi & Zhang [24] on music therapy as treatment for aphasia suggests the basal ganglia facilitate language only insofar as they handle rhythm and beat processing, temporal prediction, and the execution of motor programs.

As with the basal ganglia, Nadeau [23] emphasized the purely computational nature of thalamic circuits involving language centers. The most likely explanation of thalamic aphasia, he writes, is diaschisis. Crosson [25] posits that cortico-thalamo-cortical connections maintain semantic representations while they are compared to lexical items generated in language cortex. This comparison by the thalamus generates an error signal that allows the interface between semantic and lexical mechanisms to be fine-tuned until the desired match is achieved and eventually sent to other cortical areas. However, the literature contains discrepant reports of the symptoms of individuals with thalamic aphasia. De Witte et al. [26] found, for example, that 14 of 32 (43.8%) patients with acute LH thalamic lesions presented with language comprehension problems (although only 3 of these cases were severe). Strikingly, the authors report that 69% of patients (n = 26) with LH thalamic lesions had moderate to severe naming problems. In a study by Rangus et al. [27], however, the authors found that when present, language deficits secondary to LH thalamic lesions were mild; 96% showed fluent, spontaneous speech. Moreover, naming deficits were only found in 19% of patients with LH thalamic lesions, and auditory and verbal comprehension were intact.

The variation in the type and severity of language deficits after subcortical stroke is likely due to a variety of factors. The link between greater lesion volume and poorer language abilities in acute *cortical* aphasia has been consistently reported [4], but such a link has only seen some support in subcortical aphasia patient populations [28]. In acute left hemisphere stroke survivors with a history of prior stroke, Goldberg et al. [29] found that the total amount of brain damage incurred by strokes was a stronger predictor of acute language impairments than a categorical stroke history variable (i.e., history versus no history of prior stroke). Olsen et al. [30], however, argued that infarcted subcortical tissue does not itself account for subcortical aphasia. They instead implicated cortical hypoperfusion (i.e., the deprivation of oxygenated blood flow to cerebral cortex due to artery occlusion) [31] as the primary driver of language impairment after subcortical stroke. Further, Hillis et al. [32] demonstrated that the reversal of cortical hypoperfusion is associated with the resolution of aphasia. They also reported that cortical hypoperfusion better predicted presence of aphasia than non-thalamic subcortical lesion volume did. The case for hypoperfusion as the primary cause of subcortical aphasia was strengthened by another study by Hillis et al. [33], who found that aphasia classifications (Broca’s, Wernicke’s, Global, etc.) were linked to hypoperfusion in specific regions of the cerebral cortex. In accordance with these findings, Choi et al. [34] found cortical hypoperfusion in all of their aphasic participants (n = 15). In their study, the severity of aphasia correlated positively with the extent of cortical hypoperfusion. More recently, however, Sebastian et al. [35] found that aphasia can occur without hypoperfusion in left thalamic stroke, indicating that lesion location (e.g., basal ganglia vs. thalamus) and hemispheric laterality (e.g., left vs. right hemisphere) also play a role in the degree of language impairment.

There are other potential neural causes of subcortical aphasia, such as the co-occurrence of subcortical stroke and symptoms of small vessel disease and prior subcortical ischemia [36]. In particular, leukoaraiosis is a form of white matter disease characterized by white matter dysfunction likely due to perfusion disturbances within arterioles that perforate the deep brain structures [37]. These diffuse hyperintensities are associated with an increased risk of cognitive impairment [38,39], and their prevalence increases with age, especially after age 60 [37,40,41]. Leukoaraiosis is more prevalent in males [42] and has been linked to poor outcomes in acute cortical stroke [43,44], including a 4.3-fold increase in chronic decline of language abilities [45]. In addition, leukoaraiosis severity is predictive of infarct volume [46], infarct growth [47], and response to language therapy in cortical stroke [48]. Whether these trends exist in subcortical stroke populations is unclear.

The present study aimed to disentangle the independent contributions of each of these factors (i.e., lesion volume and location, hypoperfusion, leukoaraiosis, prior infarcts) to language impairments in acute (less than 10 days post-stroke onset) left hemisphere subcortical stroke. We hypothesized that greater hypoperfusion, more severe leukoaraiosis, and higher total lesion volume (including damaged tissue from prior subcortical stroke) would serve as strong negative predictors of acute language abilities. As a precursor to our main aim, we also determined relationships between stroke factors and demographic variables such as age and sex.

The importance of determining which of these factors contribute to subcortical aphasia is twofold. First, characterizing subcortical participation in language functions (e.g., lexical-semantic processing), if any, will deepen our understanding of these structures beyond their well-characterized roles in relaying sensory and motoric cortical inputs and outputs. Second, the present study may guide clinicians’ decisions to look beyond infarct volume for predictors of acute subcortical aphasia. That is, if leukoaraiosis, hypoperfusion, and/or a history of prior stroke can independently account for the incidence or severity of subcortical aphasia, those findings may form a more complete clinical picture of subcortical stroke during its arguably most critical window.

## Materials and methods

### Participants

We retrospectively reviewed 1043 records of individuals who were admitted to Johns Hopkins Hospital or Bayview Medical Center between 2002 and 2019 and recruited as part of a larger study aimed at investigating left hemisphere stroke recovery. The initial sample included 80 individuals (36 women, mean age = 55.7 years, SD = 14.5 years) who were confirmed to have an acute ischemic LH subcortical stroke *without* cortical involvement or acute lesion elsewhere in the brain (e.g., brainstem, cerebellum). Analyses of the relationships between demographic and stroke/brain variables were done with this broader sample. Our final sample, however, comprised 66 individuals (29 women, mean age = 57.6 years, SD = 13.0 years) who had sufficient language data for analysis. All analyses related to language performance (i.e., those using language summary z-scores) were done with this subsample.

The average time interval between the stroke date and administration of language testing was 1.61 (1.45) days, while the average gap between clinical imaging acquisition and language battery administration was 0.95 (1.08) days. A subset of our sample (n = 28) had prior strokes restricted to subcortical structures in one or both hemispheres (left = 6, right = 8, bilateral = 14) in addition to their acute LH subcortical lesion. Patients with underlying neuropathology (e.g., Alzheimer’s disease) besides stroke were excluded so that any language impairments present could be attributed to stroke and other variables of interest (e.g., leukoaraiosis) and not to other causes. No subjects with hemorrhagic stroke were included in the sample. For further information about subjects’ demographics, stroke etiology, and risk factors, see S1 Table. Ethics approval was obtained from the Johns Hopkins University institutional review board, and written informed consent was provided by all participants prior to enrolling in the study.

### Neuroimaging data

Upon their acute hospitalization, each patient underwent a clinical magnetic resonance imaging (MRI) protocol that included diffusion-weighted (DWI), T2-weighted, and fluid-attenuated inversion recovery (FLAIR) sequences. Because MRI acquisition protocols varied throughout the 17 years across which our participants were seen, we report the parameters for each patient and scan type in S2–S4 Tables. The average time interval between the acute stroke and clinical imaging acquisition was 0.95 (1.08) days.

Using MRIcron [49], trained technicians manually delineated acute lesions slice-by-slice on DWI scans, which are highly sensitive to stroke lesions in the acute phase [32]. Similarly, chronic lesions were manually delineated on T2-weighted imaging for the 28 individuals with prior subcortical involvement. From these tracings, acute lesion volume, total lesion volume (acute + chronic volumes for participants with prior stroke), and the percentage of damaged tissue in subcortical regions of interest (ROIs) were calculated using NiiStat [50]. Subcortical ROIs extracted from the Johns Hopkins University atlas [51] included subparts of the basal ganglia (caudate nucleus, putamen, globus pallidus), thalamus, superior and posterior corona radiata, anterior and posterior limbs of the internal capsule, and external capsule.

The severity of leukoaraiosis both ipsi- and contralateral to the acute LH infarct was visually assessed on T2-weighted MRI imaging and rated using the Fazekas scale [52], which comprises two qualitative subscales. The first captures periventricular hyperintensities (PVH), found near the horns of the lateral ventricles, and is scored from 0 (no hyperintensities) to 3 (irregular hyperintensities extending into the deep white matter). The second subscale evaluates the severity of deep white matter hyperintensities (DWMH) and is also scored from 0 (no hyperintensities) to 3 (large confluent hyperintense areas). PVH and DWMH subscale ratings were made independently for the left and right hemisphere by two independent, trained raters (M.S. and E.M.). Consensus ratings were given when ratings by M.S. and E.M. differed by more than one point. Because chronic stroke lesions also appear hyperintense on T2-weighted images, the manual tracing of the prior stroke lesion was overlaid on the T2-weighted image while the leukoaraiosis ratings were completed for the 28 individuals with a history of subcortical stroke recurrence.

To prepare the Fazekas data for analysis, we next compared left and right hemisphere (RH) Fazekas ratings using Wilcoxon rank sum tests. Leukoaraiosis is often symmetrical between hemispheres, and indeed, we found no significant hemispheric differences for either PVH (W = 3307, *P* = 0.697) or DWMH (W = 3063, *P* = 0.617) ratings. In contrast, within each hemisphere, PVH ratings significantly differed from DWMH ratings (LH: W = 3943, *P* = 0.007; RH: 3977, *P* = 0.004). Therefore, we averaged the ratings from each hemisphere to generate a single PVH and single DMWH rating for each participant. Inter-rater reliability was calculated according to Cohen’s weighted kappa (κ_w_ = 0.676, *P* < 0.001 for PVH scores; κ_w_ = 0.457, *P* < 0.001 for DWMH scores).

**Fig 1:**
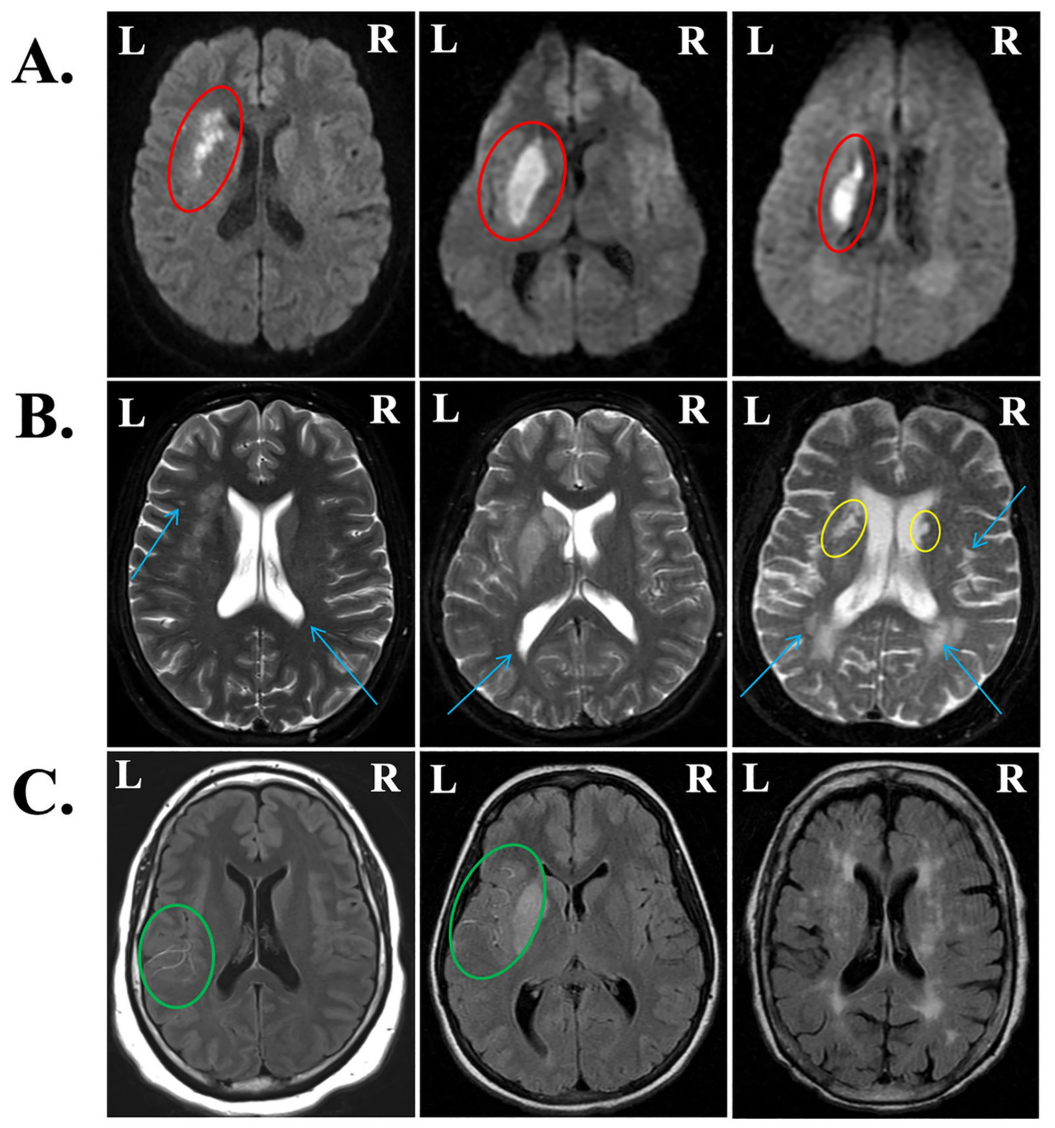
Representative images from patients with varying acute and chronic infarct volumes, leukoaraiosis, and hypoperfusion. (A) Diffusion-weighted images (DWI) display acute lesions (red ovals). (B) T2-weighted scans were used to visually score leukoaraiosis on the Fazekas scale (blue arrows) from 0 to 3 and visualize chronic infarcts where present (yellow ovals). From left to right, periventricular hyperintensities (PVH) were scored as 1, 1, and 3; deep white matter hyperintensities (DWMH) were scored as 1, 0, and 2. (C) FLAIR hyperintense vessels (FHV) are indicative of hypoperfusion in areas surrounding infarcted tissue (green ovals). From left to right, FHV scores were 5, 7, and 0 out of 12.

Regions of hypoperfusion often extend beyond the lesioned tissue itself, which offers additional information about the extent of possible language deficits [53]. In clinical sequences, perfusion-weighted imaging (PWI) is often used to quantify hypoperfusion, yet the limited availability of perfusion data in our sample made a PWI approach to assessing hypoperfusion untenable. Notably, arteries supplying hypoperfused areas of the brain appear bright (hyperintense) on FLAIR imaging due to slow blood flow [54]. Therefore, we evaluated FLAIR scans for hyperintense vessels in each of the vascular territories that supply the brain with perfused blood. Reyes et al. [55] found that FLAIR hyperintense vessels (FHV) are a reliable surrogate for PWI data and accurately reflect the burden of hypoperfusion in the acute phase. Recently, Bunker at al. validated the use of FHV scores in lieu of PWI in correlational analyses with language metrics in acute ischemic stroke [56]. We employed the NIH-FHV scoring system used by Reyes et al. [55], which rates the number of hyperintense vessels per axial slice in each of six vascular territories in the affected (left) hemisphere. The vascular territories assessed were those of the anterior cerebral artery, posterior cerebral artery, and the frontal, temporal, parietal, and insular divisions of the middle cerebral artery. In each territory, a score of 0 denotes no FHVs in any slice and a score of 2 denotes ≥ 3 FHVs per slice or FHVs present in ≥ 3 axial slices. To index overall hypoperfusion, we summed all territory FHV ratings to generate a single total FHV measure. Total FHV scores for each patient (78/80 received FLAIR imaging) could range from 0 to 12. Inter-rater reliability for the total FHV rating between the two trained raters (M.S. and A.W.) was calculated according to Cohen’s weighted kappa (κ_w_ = 0.934, *P* < 0.001). Notably, the distribution of total FHV scores was heavily skewed to the lower end of the scale, with 47 participants with no FHVs (i.e., scores of 0). As such, the FHV rating was transformed into a dichotomous variable such that 0 reflected no hypoperfusion (i.e., 0 FHVs) and 1 reflected some degree of hypoperfusion (i.e., ≥ 1 FHV). See Fig 1 for a visualization of acute lesions on DWI (Fig 1A), visualizations of prior lesions and leukoaraiosis on T2-weighted images (Fig 1B), and hyperintense vessels on FLAIR (Fig 1C).

Because MRI acquisition protocols varied throughout the years, we conducted several tests to determine whether variability in scan parameters (i.e., magnet field strength, voxel size, and slice thickness) influenced stroke measures of interest (i.e., lesion volume, leukoaraiosis ratings, and hypoperfusion ratings) in meaningful ways. Non-parametric statistics (i.e., Wilcoxon rank sum tests, Spearman correlations) were used due to the non-normal distribution of MRI variables. Magnet field strength (either 1.5T or 3T) and voxel size (in mm^3^) were considered proxies of scan resolution. We hypothesized that differences in scan resolution would most likely affect the precision of acute stroke lesion tracings (performed on DWI), prior stroke lesion tracings (performed on T2-weighted scans), and percent damage to regions of interest (likely driven, if anything, by DWI voxel size). Similarly, we hypothesized that scan resolution may have affected the clarity of white matter hyperintensities visible on T2-weighted scans, thereby affecting our ability to accurately rate leukoaraiosis severity and extent. Indexing hypoperfusion via the FHV schema involved determining the number of consecutive brain slices with hyperintense vessels; therefore, we predicted that of all MRI variables, FLAIR slice thickness (in mm) would most likely influence the precision of hypoperfusion ratings. Nonetheless, as shown in S5 Table, there were no significant relationships between any MRI and stroke variables even without correction for multiple comparisons. Therefore, we did not control for any scan parameters in the main study analyses.

### Language assessments

Because our sample draws from patient data that spans 17 years (2002–2019), participants were administered a variety of language assessments. Participants received either the Boston Diagnostic Aphasia Examination (BDAE, n = 15) [57], Western Aphasia Battery-Revised (WAB-R, n = 25) [58], or an in-house lexical battery (LB, n = 30). While the LB was in-house, it has been normed and employed in published work [32,59,60]. With every patient for whom we gathered language data, the complete battery was attempted, even if the patient was not able to complete every task. These batteries, while assessing similar components of language ability, employ unique tasks and scoring systems. For this reason, we did not statistically examine language domains, but equivalent subtests were extracted from each battery to index auditory comprehension, naming, and verbal expression skills (see S6 Table) and generate a language summary z-score, i.e., a single measure of overall language deficit severity for each participant. The z-scores were calculated using the formula z = (x-μ)/σ, where x corresponds to each patient’s raw subtest score, μ corresponds to the sample’s subtest-specific mean score, and σ corresponds to the sample’s subtest-specific standard deviation. The final z-score for each participant was generated by averaging all subtest-specific z-scores. We believe language summary z-scores are a good general reflection of overall language deficits regardless of the battery used. Such summary scores have been employed with success in recent work [29,61,62].

Our original research question relied on relating the relevant imaging variables to the *presence* of aphasia, so we first generated a binary aphasia variable to report descriptive statistics of demographics and stroke variables across the entire group of patients and by aphasia status (see Table 1). Kertesz et al. [58] established an aphasia quotient (AQ) of 93.8 or less (out of 100) to denote aphasic status on the WAB-R, while Hillis et al. [32] demarcated a score of 89% or below on the LB to denote aphasic status. The z-scores corresponding to the published aphasia cutoffs on the WAB-R and LB were similar (WAB-R z-score = -0.308, LB z-score = -0.336). No published cutoff for aphasic/non-aphasic status is readily available for the BDAE, so the average of the z-scores corresponding to the LB and WAB cutoffs was used to establish the aphasic/non-aphasic cutoff for the BDAE (z = -0.322). While framing our questions in terms of presence/absence of aphasia aligns with our initial theoretical framework, we elected to retain the language summary z-scores as continuous variables in our statistical tests for two reasons. First, the distribution of the language summary z-scores was not bimodal (which would suggest two discrete groups of patients with versus without aphasia) but instead was highly left-skewed with many more patients demonstrating mild compared to severe language deficits. Second, many patients had scores close to the cutoff for each assessment, making a definitive aphasia status classification unreliable. We accounted for the non-normal distribution of our data via the use of non-parametric statistics, as described below.

**Table 1:** Demographic and stroke variables compared between aphasic and non-aphasic participants.

Measure	All Patients (n = 80)	Aphasic (n = 21)	Not Aphasic (n = 45)
Age, mean (SD) in years	55.66 (14.50)	61.33 (13.85)	55.8 (12.41)
Sex, count (F/M)	36/44	8/13	19/26
Acute Lesion Volume, mean (SD) in mm^3^	3221.56 (4691.67)	4858.48 (5546.14)	1836.29 (1668.13)
Total Lesion Volume, mean (SD) in mm^3^	3828.11 (4774.46)	5773.38 (5458.95)	2411.60 (2175.08)
Multiple Strokes, count (Y/N)	52/28	10/11	13/32
% damage thalamus, mean (SD)	0.039 (0.065)	0.045 (0.088)	0.036 (0.049)
% damage external capsule, mean (SD)	0.042 (0.132)	0.066 (0.142)	0.014 (0.043)
% damage internal capsule, mean (SD)	0.064 (0.089)	0.096 (0.090)	0.045 (0.062)
% damage corona radiata, mean (SD)	0.042 (0.077)	0.052 (0.046)	0.028 (0.046)
% damage putamen (SD)	0.048 (0.139)	0.076 (0.180)	0.021 (0.048)
% damage caudate (SD)	0.050 (0.139)	0.105 (0.228)	0.024 (0.050)
% damage globus pallidus (SD)	0.031 (0.086)	0.050 (0.108)	0.017 (0.050)
Fazekas PVH (median)*	1.5	2	2
Fazekas DWMH (median)*	1	2	1
Hypoperfusion, count (Y/N)**	31/47	8/13	18/25

Notes:

*Fazekas periventricular hyperintensity (PVH) and deep white matter hyperintensity (DWMH) scores were averaged across hemispheres because preliminary analyses revealed no significant interhemispheric differences for either Fazekas subscale.

**Two participants in the “not aphasic” group did not have FLAIR images and consequently, did not have hypoperfusion ratings.

### Statistical analysis

All statistical analyses were performed using R Studio [63]. Brain variables of interest included: acute lesion volume and total lesion volume (i.e., the same value as acute lesion volume for patients without stroke recurrence and combined acute and prior stroke volumes for patients with stroke recurrence); a binary stroke history variable (i.e., 0 = no history of prior stroke, 1 = history of prior stroke) to determine whether stroke recurrence in itself is related to demographics and language outcomes; percent damage to ROIs (i.e., thalamus, subparts of the basal ganglia [putamen, caudate, and globus pallidus], corona radiata, internal capsule, and external capsule) to index lesion location; the binary FHV measure to capture hypoperfusion extent; and PVH and DWMH ratings within each hemisphere to measure leukoaraiosis. First, to determine relationships between brain variables and demographics, we conducted Wilcoxon rank sum or chi-square tests to determine if brain variables varied between sexes and Wilcoxon rank sum tests or Spearman correlations to examine relationships between brain variables and age. Education level was not among the demographic variables analyzed due to sporadic availability (present for only 30/80 participants). We corrected for multiple comparisons at a false discovery rate (FDR) of *Q* < 0.05.

We also determined if language deficit severity (per language summary z-scores) varied by age or sex using Spearman correlation and Wilcoxon rank sum tests, respectively. To address our main aim, we examined relationships between language summary z-scores and brain variables of interest using Spearman correlations (for continuous and ordinal variables) and Wilcoxon rank sum tests (for categorical variables), correcting for multiple comparisons using the FDR correction of *Q <* 0.05 across all tests. Finally, to determine the relative contributions of each variable on presence of language impairment severity, we conducted a Least Absolute Shrinkage and Selection Operator (LASSO) regression including all predictors that significantly related to overall language deficits via bivariate relationships as the independent variables and language summary z-score as the dependent variable. LASSO regression is particularly useful when variables exhibit high multicollinearity (as is the case with brain data), as it uses L1 regularization to shrink certain parameter estimates to zero, resulting in an optimally predictive, sparse model [64]. The days between stroke incidence and language testing was also included in this model, as changes in neurological function occur rapidly in the acute window, sometimes on the order of days or hours.

## Results

### Demographic relationships

In the full sample (n = 80), older age was linked to more severe leukoaraiosis in the periventricular white matter (*r* = 0.553, *P* < 0.001, *Q* < 0.001) and deep white matter (*r* = 0.534, *P* < 0.001, *Q* < 0.001). People with a history of multiple strokes were also older than individuals with a single stroke, although this finding did not survive multiple comparison correction (*W* = 481.0, *P* = 0.013, *Q* = 0.060). No other significant relationships between demographic and stroke/language variables were found (see S7 Table).

### Relationships between brain variables and language deficit severity

A subset of 66 patients with language summary z-scores were included in most analyses addressing the main study aim. Of these, 21 patients were designated as aphasic (31.8%) according to the aforementioned cutoffs, and 45 were not aphasic. Participants without FLAIR scans (n = 2) were excluded from the analyses involving hypoperfusion data. See Table 1 for descriptive statistics of demographic and brain variables across the entire sample and the subsample with language data, split by aphasia status.

**Fig 2:**
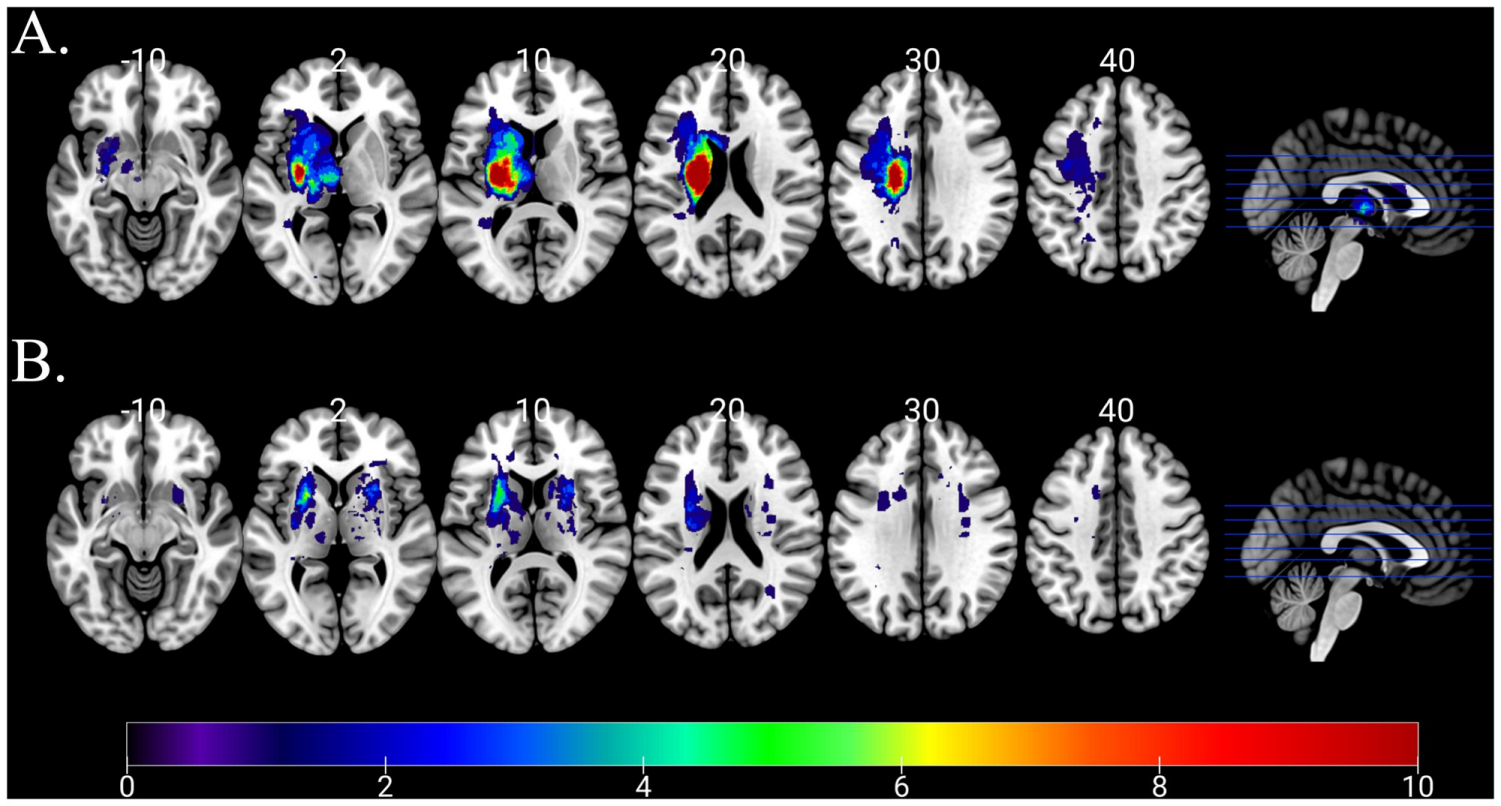
Lesion overlap maps of A) acute stroke lesions and B) prior stroke lesions. The color bar applies to both parts of the figure and indicates the number of patients whose lesions overlap for a given voxel.

More severe acute language impairments were related to larger acute lesion volumes (*r* = -0.439, *P* < 0.001, *Q* = 0.002) as well as larger *total* lesion volumes (*r* = -0.379, *P* = 0.002, *Q* = 0.004), which included both the acute LH and prior subcortical lesions. Yet, a history of prior stroke did not significantly influence global language deficits in our sample (*W* = 543.0, *P* = 0.518, *Q* = 0.561). An overlap map for both acute and chronic lesions in our sample is shown in Fig 2. Of note, the vast majority of our participants’ lesions were quite small. Only 12/80 participants had acute lesion loads greater than 5000 mm^3^, and only 17/80 of them had *total* lesion loads greater than 5000 mm^3^. Lesser lesion loads across the basal ganglia, thalamus, and entire subcortical white matter perhaps account for the high lesion variability in our sample. We might expect higher overlap in a sample of patients with large cortical lesions, but we excluded patients with any cortical damage. Despite the small lesion volumes for many patients, more severe language impairments were significantly related to greater percent damage to the corona radiata (*r* = -0.424, *P* < 0.001, *Q* = 0.002), external capsule (*r* = -0.366, *P* = 0.003, *Q* = 0.005), internal capsule (*r* = -0.416, *P* < 0.001, *Q* = 0.002), putamen (*r* = -0.402, *P* < 0.001, *Q* = 0.003), caudate (*r* = -0.288, *P* = 0.019, *Q* = 0.035), and globus pallidus (*r* = -0.268, *P* = 0.030, *Q* = 0.048) but not to the thalamus (*r* = 0.188, *P* = 0.132, *Q* = 0.190). We found no significant relationships between language summary z-scores and leukoaraiosis by either Fazekas subscale (PVH: *r* = -0.145, *P* = 0.244, *Q* = 0.317; DWMH: *r* = -0.111, *P* = 0.374, *Q* = 0.442) or presence of hypoperfusion (*W* = 534.5, *P* = 0.584, *Q* = 0.584).

### LASSO regression predicting language deficit severity from significant univariate predictors

Last, we conducted a LASSO regression that included all brain predictors that were related to language summary z-scores via previous tests before correction—including total lesion volume and percent damage to the corona radiata, external capsule, internal capsule, putamen, caudate, and globus pallidus—as well as nuisance regressors (i.e., days between testing and stroke onset, age). Within this model, only total lesion volume was significantly related to global language impairment severity (*P* = 0.043). See Table 2 for the full model results.

**Table 2:** LASSO regression predicting language summary z-scores from significant univariate predictors.

Variable	Est.	Z-score	95% CI	*P*
Time between Stroke & Testing (in days)	0.011	0.116	-3.251, 0.112	0.916
Age (in years)	-0.081	-0.834	-0.582, 0.364	0.325
Total Lesion Volume (in mm^3^)	-0.329	-2.002	-0.94, 0.059	0.043*
% Damage to External Capsule	0.085	0.362	-2.748, 0.582	0.742
% Damage to Internal Capsule	0.235	1.587	-0.18, 0.527	0.117
% Damage to Corona Radiata	-0.225	-1.851	-0.461, 0.451	0.263
% Damage to Putamen	-0.367	-1.161	-1.746, 2.883	0.529
% Damage to Caudate	-0.076	-0.388	-0.602, 1.77	0.672
% Damage to Globus Pallidus	-0.248	-1.744	-0.779, 0.115	0.076

**Notes**: Lambda = 0.058 with alpha = 0.05.

* denotes significance at *P<*0.05.

These results suggest that while subcortical stroke can cause aphasia, it often does not—and when it does, the deficits tend to be mild and linked to lesion volume. Despite having 21 individuals who were classified as aphasic, most had z-scores that were close to their respective cutoff and presented with mild deficits. The significant differences before correction were driven by three patients with marked language deficits (z-scores 1.5 or more standard deviations below the sample mean). Their imaging and language assessment profiles were examined further.

### Patients with severe language deficits

#### Patient 1

**Fig 3:**
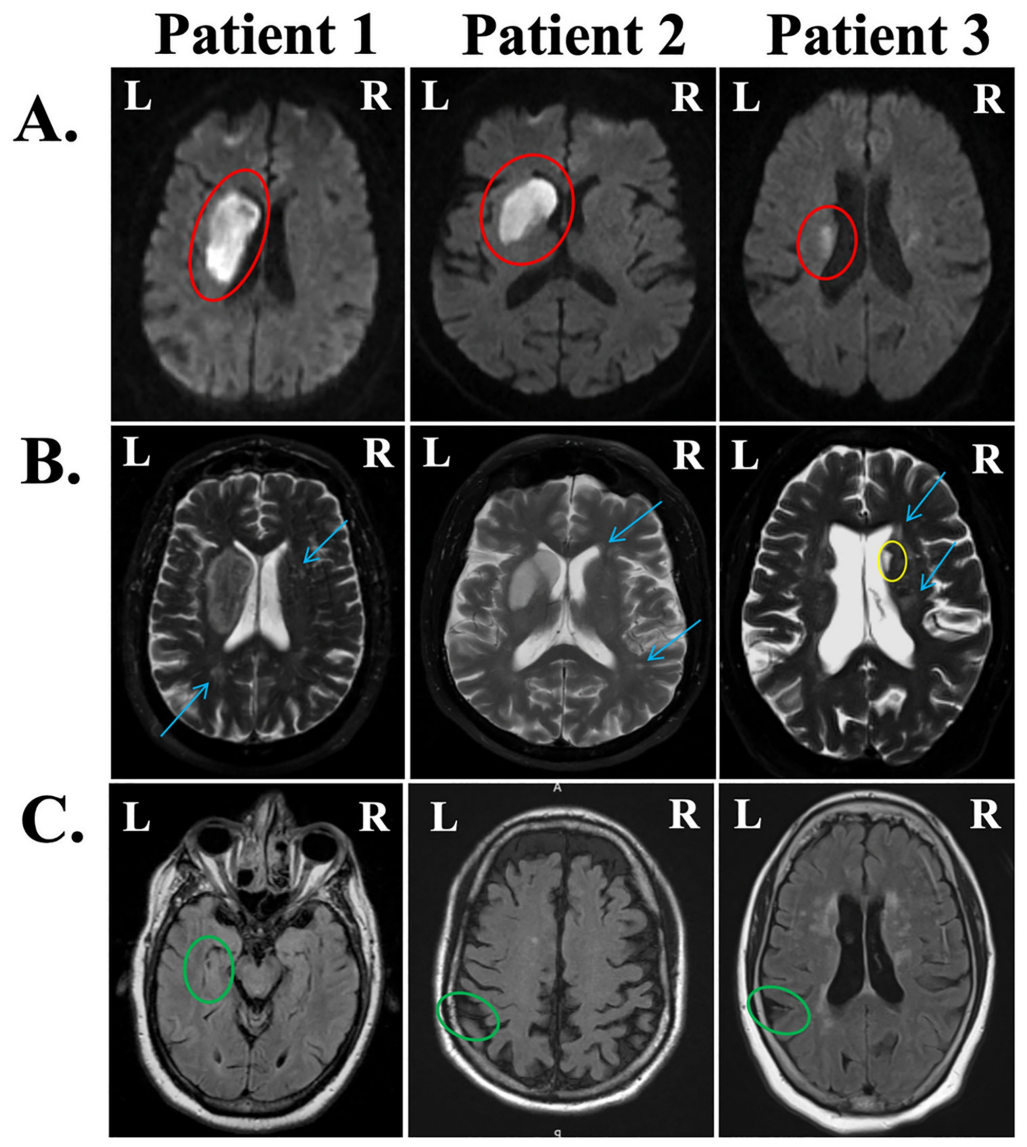
Clinical images from the three patients with marked language deficits in the acute phase. (A) Diffusion-weighted images (DWI) display acute lesions (red ovals). Patients 1 and 2 had lesions restricted to the basal ganglia, and Patient 3 had lesions to the basal ganglia and internal capsule. (B) T2-weighted scans were used to visually score leukoaraiosis on the Fazekas scale (blue arrows) from 0 to 3 and visualize chronic infarcts where present (yellow ovals). From left to right, periventricular hyperintensities (PVH) were scored as 2, 1, and 2; deep white matter hyperintensities (DWMH) were scored as 1, 1, and 2. (C) FLAIR hyperintense vessels (FHV) are indicative of hypoperfusion in areas surrounding infarcted tissue (green ovals). Only Patient 3 had a prior stroke. All three patients received FHV scores of 1 out of 12.

Patient 1 was a 70-year-old man with no prior strokes who presented with total anarthria (absence of speech). This patient used a communication board and was able to nod or shake his head. He received the WAB-R and performed 2.7 standard deviations below the mean (AQ = 55.4). Patient 1’s auditory comprehension for simple yes/no questions was intact, and single-word auditory comprehension was also mostly intact (54/60). Meanwhile, sentence comprehension for complex syntactic structures, notably those of multistep commands, was impaired. The patient’s forward word span was mildly impaired, but backwards span was severely impaired. Patient 1’s DWI revealed a large LH basal ganglia lesion (corpus striatum, 20,090 mm^3^) that extended into the periventricular white matter. Periventricular leukoaraiosis was rated 2/3 and DWMH was rated 1/3. Patient 1 was given an FHV score of 1/12 as a measure of hypoperfusion. See Fig 3A for a visualization of Patient 1’s imaging data.

#### Patient 2

Patient 2 was a 66-year-old man with no prior strokes who presented with severely impaired word fluency. For example, when asked to name as many animals as possible, Patient 2 said “Pork and beans, camel, bacon, pork, a whole bunch of animals that eat pork.” The patient was hospitalized for nearly a month after his stroke due to a fall with possible loss of consciousness (which co-occurred with the stroke). He received the WAB-R and performed 3.055 standard deviations below the mean (AQ = 76.9). Patient 2 made unique word errors in expressive naming and sentence completion tasks; he used off-target, unrelated words, and also produced some semantic paraphasias (e.g., using *envelope* for *mail*, *May* for *December*). As with Patient 1, Patient 2 was impaired with multistep commands (55/80). This patient’s stroke was localized to the left basal ganglia (caudate and lentiform nuclei, 16.871 mm^3^). Both PVH and DWMH scores were 1/3, and hypoperfusion was rated 1/12. See Fig 3B for a visualization of Patient 2’s imaging data.

#### Patient 3

Finally, Patient 3 was a 59-year-old woman admitted to Johns Hopkins Hospital for previous symptomatic infarcts, presenting with dysarthria but not aphasia. Patient 3 had bilateral basal ganglia involvement (right striatocapsular region and both caudate heads). She presented with left-sided weakness, and received the BDAE, performing 2.2 standard deviations below the mean. This patient’s auditory comprehension was intact (15/16), and she was able to follow multistep commands well. Patient 3 was able to produce some single words, but rarely surpassed one-word phrase lengths (1/7 grammatical form, 2/7 articulatory agility on the Rating Scale Profile of Speech Characteristics within the BDAE). In addition, Patient 3’s sentence repetition was poor (0-20^th^ percentile). Patient 3’s DWI revealed an acute LH infarct localized to the basal ganglia and internal capsule (1523 mm^3^). The patient’s prior bilateral subcortical lesions totaled 776 mm^3^, for a total lesion volume of 2299 mm^3^. Both PVH and DWMH scores were 2/3, so perhaps the severity of this patient’s leukoaraiosis contributed to her deficits. The patient’s total hypoperfusion was rated 1/12. See Fig 3C for a visualization of Patient 3’s imaging data. Comprehensive language data for all three patients are reported in S8 Table.

## Discussion

In this study, we investigated the contribution of multiple brain metrics on the severity of language impairment in acute left hemisphere subcortical stroke. Overall, language performance was related to acute/total lesion volume and greater damage to many of our brain regions of interest, but not to leukoaraiosis severity, hypoperfusion, or a history of prior stroke. Ultimately, however, the only significant predictor of language performance was the total lesion volume. The majority null findings are important from a clinical and scientific standpoint in this population. Below, we address each finding in greater detail and situate the results in the context of previous literature.

### The frequency and severity of acute subcortical aphasia

In our study, we found that 68.2% of patients were not aphasic, whereas only 31.8% were aphasic according to our language summary z-scores cutoffs (see Table 1). The prevalence of aphasia in acute subcortical stroke has varied widely. Hillis et al. [32] reported aphasia in 25 out of 37 patients (67.6%) with acute strokes restricted to subcortical structures. In a later study, Hillis et al. [33] reported an aphasia frequency of 54.1% in a patient corpus of 24 patients, although 16 of these patients were also included in the earlier study. Interestingly, another study by Choi et al. [34] found that 15 of 16 patients (93.8%), all of whom had acute left striatocapsular strokes and were administered the Korean version of the WAB, were aphasic. Olsen et al. [30], on the other hand, found that only 8 of 18 patients (44.4%) with acute subcortical LH lesions were aphasic. That the majority of patients in their study did not even exhibit minimal speech-language deficits to meet the criteria for mild aphasia suggests a high proportion of acute LH subcortical stroke patients whose language is completely intact.

One key difference across these studies, including our own, is the use of different language batteries and scales to measure aphasia. Both studies by Hillis et al. [32,33] used the lexical battery, and the later study also used the BDAE for measures of repetition, auditory comprehension, and naming. Choi et al. [34] instead used the WAB, which measures spontaneous speech generation, auditory comprehension, repetition, and naming. Our study used data from an in-house lexical battery, BDAE, and the WAB, and extracted measures of auditory comprehension, naming, and verbal expression skills. Meanwhile, Olsen et al. [30] used an aphasia severity scale proposed by Goodglass et al. [65], which assigns a score from 0 to 3 based on qualitative language outcomes. In addition to testing different domains of language, the different batteries may have varying sensitivities to aphasia. Of note in our study, however, is the largest patient sample, which is perhaps more representative of the acute LH subcortical stroke population than earlier studies.

Accounts of the severity of LH subcortical aphasia are similarly inconsistent. Only three patients in our sample (4.5%) presented with severe language deficits (language z-scores more than 1.5 standard deviations below the mean). Many subjects who *were* aphasic bordered established cutoffs for aphasia in their respective language batteries. For example, six of eight aphasic patients who received the WAB scored above 90 but below the aphasia cutoff of 93.8. Of the eight aphasic patients in the study by Olsen et al. [30], only one had severe aphasia, whereas three had mild aphasia and the last three had moderate aphasia. We are led to believe, then, that when language deficits do occur after LH subcortical stroke, they are not often severe. While the distributions of severe versus borderline cases of aphasia in the other aforementioned studies are not reported, the authors of the later study by Hillis et al. [33] acknowledge inconsistencies in aphasia severity due to subcortical infarcts.

### Relationship between lesion location and aphasia

#### Thalamus

The link between overall brain damage, captured by total lesion volume, and deficit severity is well-established. Relationships between language deficits and specific lesion sites, however, are more debated. Numerous functional imaging and clinical studies have implicated thalamic involvement in language processing [13,66–69]—and left thalamic damage has previously been linked to language deficits, but our results do not corroborate this link. Notably, the only brain ROI whose extent of damage was *not* initially correlated with the language summary z-scores was the thalamus. Nevertheless, recent accounts of the incidence and severity of thalamic aphasia are mixed.

Hillis et al. [32], for example, found that 0 of 5 patients with isolated LH thalamic strokes were aphasic. On the other hand, Karussis et al. [70] reported aphasia in 88% (n = 8) of acute left thalamic stroke patients, and a meta-analysis by De Witte et al. [26] reported aphasia in 64% (n = 11) of patients with acute left thalamic lesions. Further, Sebastian et al. [35] found that among a sample of 10 patients with isolated left hemisphere thalamic stroke, half were aphasic. Recently, a study by Rangus et al. [27] found that 48% (n = 31) of patients with isolated acute LH thalamic lesions met their diagnostic criteria for aphasia. However, naming and auditory comprehension—which informed our language summary z-scores—were largely intact in their participants.

In addition to the use of different language measures, the discordance between our study and those linking thalamic damage to language deficits might be due to our sample’s low proportion of thalamic stroke patients, who themselves did not present with large thalamic lesions. Only 13 patients out of 80 (16.4%) had greater than 10% damage to the left thalamus (range = 0–31.6%). Of these 13 patients with significant thalamic damage, only four were aphasic (30.7%). Moreover, of the 21 aphasic patients in our sample, only four (19%) had greater than 10% damage to the left thalamus. Overall, then, the frequency of thalamic lesions among purely subcortical lesions was low in our study, as was the frequency of aphasia among patients with significant thalamic damage.

The nucleus of the thalamus that is infarcted is almost certainly important in determining whether or not aphasia is present. The various nuclei of the thalamus are known to be involved in distinct functions (e.g. attention, somatosensory function, motor relay, limbic functions). However, it is difficult to localize the specific nucleus of the thalamus on clinical MRI. Doing so remains an important future direction for research involving high resolution research MRI in which segmentation of thalamic nuclei is feasible.

#### Basal ganglia

As with the thalamus, there is little consensus surrounding the role of the basal ganglia in language. In our sample, language performance was negatively correlated with greater percent damage to the caudate, putamen, and globus pallidus, even after correction for multiple tests. Basal ganglia damage was not, however, a significant predictor of language impairment severity in our final regression. A recent meta-analysis by Radanovic & Mansur [8] illuminates the state of the literature in characterizing the frequency and phenotype of basal ganglia stroke. Their review generated 180 patients from 57 separate studies with acute left hemisphere lesions restricted to the basal ganglia, with or without damage to the internal capsule. Overall, 67.3% had language deficits of some sort, although this incidence varied greatly depending on the structure affected. For example, patients whose damage was isolated to the caudate (n = 24), putamen (n = 31), or globus pallidus (n = 4) had language disturbances at a rate of 41.7%, 80.7%, and 0%, respectively. The production of paraphasias was linked to damage to the putamen (odds ratio (OR) of 2.71), whereas repetition impairments and nonfluent aphasia were less associated with caudate lesions (OR of 0.17 and 0.28, respectively).

When multiple basal ganglia are affected, with or without involvement of the adjacent white matter, accounting for the clinical picture of these patients becomes more difficult. Radanovic & Mansur [8] also found that on their own, lesions to the putamen were only associated with production of paraphasias, and pallidal lesions alone were not associated with any language symptoms. However, in patients with strokes isolated to the lentiform nucleus (i.e., the putamen and globus pallidus), the authors report a higher likelihood of repetition impairment, comprehension deficit, and nonfluent aphasia (OR of 5.78, 3.50, 3.23, respectively). Further, the authors found that neither striatal (affecting the caudate and putamen) nor striatopallidal lesions (affecting the caudate, putamen, and globus pallidus) were associated with language symptoms.

Within our sample of 80 patients, significant damage (greater than 10%) to structures of the basal ganglia was uncommon. When such damage *did* occur, it was rarely isolated to a single structure, and rarely was it exclusive of the surrounding white matter. Few patients had greater than ten percent damage to the caudate nucleus (10.0%), putamen (11.3%), and globus pallidus (8.8%). Had our sample contained more patients with significant and exclusive basal ganglia involvement, it is not clear that our results would change, as our language data lacked measures of repetition—one of the few components of language associated with lesions isolated to a single structure. For the most part, taking larger subsets of basal ganglia structures, with or without inclusion of the surrounding white matter, does not reveal decisive clinicoanatomical correlations with language symptoms. In those cases where particular subsets of structures *do* reveal characteristic language symptoms, the present study was unable to capture those relationships, due in large part to the scarcity of patients with significant basal ganglia involvement and the lack of repetition data.

### Leukoaraiosis

In our study, we found that more extensive leukoaraiosis severity was related to older age but not to language performance after subcortical stroke. We did not corroborate findings that leukoaraiosis is more prevalent in men [42]. The finding that greater leukoaraiosis severity is linked with greater age has considerable support in the literature. The Leukoaraiosis and Disability (LADIS) study examined the major determinants of leukoaraiosis severity and found that age, frequency of hypertension, and prior stroke history increased with leukoaraiosis severity as measured by the Fazekas scale [71]. This study of 639 patients did not focus on stroke patients specifically. In a study of neglect performance in acute right hemisphere stroke, Bahrainwala et al. [72] reported significantly higher age among patients (n = 205) with severe leukoaraiosis compared to patients with milder leukaraiosis, as measured by the Cardiovascular Health Study (CHS) rating scale from 0–9 [73]. More recently, a meta-analysis by Vedala et al. [41] found using the Wahlund leukoaraiosis scale, which accounts for hemispheric differences as well as regional differences, that leukoaraiosis was most severe in older patients [74]. The authors found this to be true on both MRI (n = 186) and CT (n = 238) clinical imaging. Importantly, our concordance with previous studies on the link between age and leukoaraiosis severity validates our chosen method of measuring leukoaraiosis.

Prior studies have shown relationships between leukoaraiosis extent and the severity of post-stroke deficits in domains outside of language. For example, Arsava et al. [75] found a moderate correlation between leukoaraiosis volume and clinical outcomes at 6 months post-stroke as measured by the modified Rankin Scale (mRS), which reflects neurologic disability but not language in particular. Henninger et al. [42] and Arba et al. [43] had similar findings for 90-day outcomes, also using the mRS. A review by Fierini et al. [44] found that greater leukoaraiosis severity predicts poorer overall clinical outcomes in the acute and subacute phases.

From the acute to chronic stages of cortical stroke recovery, it appears that greater leukoaraiosis is associated with poorer global neurological outcomes. Such a relationship is not so clear for language in particular. A few studies have investigated the relationship between leukoaraiosis and language deficits in patients with aphasia due to *cortical* stroke. Wright et al. [40] examined language outcomes and found that in patients greater than three months post-stroke (well outside the acute window), leukoaraiosis severity as measured by the CHS scale was negatively correlated with object naming and word fluency performance after controlling for lesion volume. A retrospective study by Varkanitsa et al. [48] aimed to identify how leukoaraiosis factors into pre- and post-treatment language outcomes in the chronic phase of LH cortical stroke recovery. Before treatment, composite Fazekas scores were associated with poorer nonverbal executive function, but not with overall aphasia severity (per the WAB-R) or naming ability (per the Boston Naming Test) [76]. After semantic feature analysis treatment, though, Varkanitsa et al. [48] reported that higher pre-treatment DWMH negatively predicted the degree of language improvement. Under a similar study design, Wilmskoetter et al. [77] did not find a direct effect of PVH on WAB AQ (n = 48), but reported an indirect effect of PVH on AQ mediated by the number of intact long-range and short-range white matter fibers. Varkanitsa et al. [48] speculate that the discrepancy between the findings of their study and those of Wilmskoetter et al. is due to differences in leukoaraiosis severity among their participants, as well as sample size differences.

Basilakos et al. [45] highlighted what seems to be an important difference between acute and chronic aphasia as they relate to leukoaraiosis. In their sample of left MCA cortical strokes (n = 35), Fazekas scores were not predictors of WAB AQ during the acute phase. However, Fazekas scores at onset and initial aphasia severity together were predictors of AQ at follow-up ≥ 6 months post-stroke. The authors reported that more severe leukoaraiosis was associated with a 4.3-fold increase in the likelihood of language decline between the acute to chronic stages. If parallels in small vessel disease do exist between cortical and subcortical stroke, leukoaraiosis after subcortical stroke may be a better predictor of language outcomes in the chronic phase or in response to treatment than during the acute phase. This relationship is worth investigating in a sample of chronic subcortical stroke patients, especially those who receive treatment. The most salient difference, of course, between the present study and those cited above is our use of a strictly subcortical sample, whereas other studies deal either exclusively or primarily with cortical stroke patients. It is possible that the presence of leukoaraiosis only contributes to language deficits when there is cortical damage, but not if core language cortex is preserved (as is the case in subcortical stroke). So, while prior studies point to a link between white matter hyperintensities and language outcomes after LH stroke in general, it does not appear that this link extends to the narrower population of acute subcortical stroke patients. If leukoaraiosis severity *is* predictive of subcortical aphasia severity, our findings appear to rule out such a relationship in the acute phase.

### Hypoperfusion

Hypoperfusion in subcortical stroke has been found to be the driving factor in acute aphasia, but not in the present study. We found no significant relationships between language summary z-scores and the extent of hypoperfusion as measured by FHV ratings. Most participants showed little to no hypoperfusion. However, all three patients with poorest language performance were given an FHV score of 1/12, indicating that the presence of hypoperfusion might have contributed to their aphasia. In our sample, 60.2% of participants received FHV ratings of 0, while 23.1% received a score of 1 (n = 78). Only four participants received an FHV score of 5 or greater, and the maximum score of any participant was 7 out of a possible 12 points. Moreover, 13 of 21 aphasic patients had an FHV score of 0, and six aphasic patients received an FHV score of 1 or 2. The remaining two patients scored no higher than 6. However, it is not known if a FHV rating of 1 or 2 is a sensitive measure of cortical hypoperfusion.

That the majority of our sample exhibited no cortical hypoperfusion suggests cortical hypoperfusion is not common after LH acute subcortical stroke, at least as measured by FHV. There is significant support, however, for the hypothesis that cortical hypoperfusion (measured with MRI PWI or PET) is a driver of subcortical aphasia [30,32–34]. Sebastian et al. [35], however, found that aphasia after thalamic stroke was not due to cortical hypoperfusion, since four of the five aphasic patients in this study had cortical perfusion within normal limits. The role of hypoperfusion may vary depending on the location of the subcortical infarct. In a study by Hillis et al. [33], all participants suffered basal ganglia strokes—at least to the left caudate, but in some cases also the putamen, globus pallidus, and internal capsule. Similarly, all participants in a study by Choi et al. [34] had striatocapsular lesions. Studies whose participants suffered basal ganglia infarcts seem to implicate hypoperfusion as a driver of aphasia, whereas a sample of thalamic stroke patients implicates the infarcted region itself. This distinction may have been lost in our sample, which was quite heterogeneous; our study contained patients whose lesions were restricted to the thalamus, basal ganglia, and/or adjacent white matter structures, but no region was overrepresented as in the aforementioned studies.

Due to the limited availability of perfusion-weighted imaging in our sample, we captured cortical hypoperfusion differently than did previous studies. The qualitative nature of the FHV ratings and their lack of voxel-level specificity posed a challenge to our analysis. We did not confirm, using this approach, that the hypoperfusion is the primary driver of subcortical aphasia. Another difference is that Olsen et al. [30] and Hillis et al. [32,33] used more sensitive imaging measures of cortical hypoperfusion (PET or MRI PWI). Use of FVH has not been determined to be as sensitive as PWI in detecting cortical hypoperfusion, although (when present) the number of cortical hyperintense vessels on FLAIR correlates with the volume of hypoperfusion.

### Patients with severe language deficits

We identified three patients who had the most severe language impairments (identifying as 1.5 SD below the sample summary score mean). A lingering question from this discussion—and one that is relevant when considering these three individuals—is whether language deficits after subcortical stroke are due to a domain-general impairment or to direct insult to linguistic processing. When language is disrupted, we might suspect domain-general functions including (but not limited to) executive function, speech motor function, and working memory as being affected.

All three patients discussed here suffered lesions to the basal ganglia, and one extended to the internal capsule—although none had significant thalamic involvement. Perhaps, then, we cannot shed light on the role of the thalamus, if any, in processing language directly. It is worth noting, however, that the literature appears to favor a supportive, rather than direct role for *both* the thalamus and basal ganglia in language (see Introduction). If the basal ganglia provide only computational support to language, what might we expect from ischemic lesions to its structures? A reasonable answer, write Bohsali & Crosson [22], would be minor impairments in the accuracy or speed of word-finding rather than a severe deficit as is seen in cortical aphasia. This explanation accounts for the type and lower severity of language symptoms seen in our sample, and may also be consistent with the profiles of our three subjects with especially poor performance on language assessments. Perhaps even when subcortical strokes *do* result in aphasia, those impairments are driven by non-linguistic deficits, namely in executive functioning, working memory, and the motoric aspects of speech production.

Poor performance in Patient 1’s forward and backwards word span, as well as backward digit span, suggest a burden on working memory. Perhaps Patient 1’s impaired sentence comprehension, especially for complex syntactic structures and multistep commands, is linked to his working memory deficits rather than language, per se. Next, Patient 2’s off-target word choice errors and difficulty with multistep commands may indicate deficits in domain-general executive function skills, or potentially impaired semantic processing. Patient 2 had a fall at the time of his stroke, so we cannot rule out the possibility that his aphasia was due to, or exacerbated by, his fall with potential head injury. Patient 3, on the other hand, had intact comprehension but presented with severe expressive deficits. Patient 3’s poor articulatory agility and communication primarily through gestures suggest a motor speech deficit, likely indicative of severe apraxia of speech and/or a more severe dysarthria plus apraxia of speech.

From these cases and with support from the literature, poor language performance after acute LH subcortical stroke may not actually reflect language impairments per se. We are therefore unable to corroborate accounts explaining a non-motoric or non-executive linguistic processing role for the subcortical structures examined in this study, at least not in cases of severe language deficits.

### Limitations

Several elements of this study posed a challenge to our analysis and limited comparison with previous studies. For one, our participants were assessed over the course of 17 years, and different batteries were used to assess their language. Each of these batteries likely had different sensitivities to aphasia and was geared toward measuring different language functions. This necessitated the use of a summary z-score to allow comparisons across batteries. These summary z-scores, however, could have been more informative if there was more overlap in the language measures assessed by each battery. Potential trends in certain linguistic domains (e.g., semantics, phonology, syntax) and subdomains of language such as repetition could not be captured in our analyses.

Another limitation of the retrospective nature of the study and different assessment batteries was our inability to index concomitant motor speech impairments in our sample and disentangle language from motor speech concerns. While some versions of our language battery included an apraxia of speech measure, other iterations of the battery did not. For example, while clinician notes and the BDAE articulatory agility rating indicate Patient 3 had apraxia of speech, we do not have access to a specific apraxia of speech severity rating for this individual nor audio recordings of their verbal productions to rate ourselves. Beyond Patient 3, is possible that motor speech deficits influenced the language summary z-scores even in patients with less severe impairments. Future prospective studies must include both motor speech and language measures to determine these different influences on verbal output in subcortical stroke survivors.

In addition, factors like years of education were sporadically available due to the retrospective nature of our study. A prospective study would have allowed us to gather more detailed demographic information, but this would presumably come at the expense of the large sample size we achieved. Nevertheless, such factors could be mediating variables in relationships between stroke pathologic factors and aphasia status. Previous studies on subcortical aphasia often used only one battery, and were able to collect repetition data, which was not present in much of our sample. The use of different batteries also posed a challenge when comparing our results to those of previous studies. In addition, the literature does not contain an established aphasia cutoff for the BDAE, unlike the WAB-R and our in-house lexical battery (based on prior publications) [32,58].

As referenced previously, perfusion-weighted imaging and time-to-peak maps were only available for a small fraction of our study participants. Instead, we used FLAIR, which was readily available for nearly every participant, but generated a more qualitative metric for cortical hypoperfusion in the form of FHV scores. Whether our results are replicable in subjects with PWI data on record (and ideally, who received a single language battery) is an interesting avenue for further research. Similarly, the subjectivity of visual, qualitative ratings to index leukoaraiosis severity presented a challenge to this analysis and has for other authors as well. Caliguiri et al. [78] emphasized relatively low inter-rater reliability for qualitative visual rating scales of white matter hyperintensities. Regional differences in white matter disease burden beyond periventricular versus deep white matter hyperintensities could not be captured using the Fazekas scale, so perhaps further research using a voxel-level tool to quantify leukoaraiosis in specific structures is warranted.

## Conclusion

In this study, we investigated relationships between brain structure variables and the severity of language deficits in survivors of acute left hemisphere subcortical stroke. While acute/total lesion volume and percent damage to substructures of the deep white matter and basal ganglia were negatively correlated with language performance, only total lesion volume was ultimately a significant predictor of global language impairment severity. Given the variability in language deficits and imaging markers associated with such deficits, it seems likely that subcortical aphasia is a heterogeneous clinical syndrome with distinct causes across individuals. The focus of the present study on the acute phase of LH subcortical stroke motivates further investigation into longitudinal outcomes for patients with this clinical presentation (i.e., subacute, chronic phases). Whether those results accord with the results described here may indicate which phase of stroke recovery is the most important for the characterization of deficits and interventions for addressing them.

## Supporting information

S1 Table Characteristics of retrospective sample. Electronic medical records of all participants were canvassed for a significant history of atrial fibrillation (AF), coronary artery disease (CAD), diabetes mellitus (DM), hyperlipidemia (HL), hypertension (HTN), tobacco/smoking (TOB), and substance abuse (SA). Years of education, handedness, stroke etiology, and NIH stroke scale score on admission (NIHSS) were recorded when present. *SVID*: *Small vessel ischemic disease*. “X” denotes fields where no information was found in patient records.
(DOCX)

S2 Table Diffusion-weighted imaging parameters. *FOV* = field of view; *TR* = repetition time; *TE =* echo time.
(DOCX)

S3 Table T2-weighted imaging parameters. *FOV* = field of view; *TR* = repetition time; *TE =* echo time.
(DOCX)

S4 Table Fluid-attenuated inversion recovery imaging parameters. *FOV* = field of view; *TR* = repetition time; *TE =* echo time.
(DOCX)

S5 Table Relationships between MRI parameters and stroke variables. The hypoperfusion metric used was the binary variable reflecting presence of hypoperfusion versus no hypoperfusion. Wilcoxon rank sum tests or chi-square tests were used to determine if stroke variables varied by magnet field strength. Spearman correlations were used to determine possible relationships between voxel size and thickness and related stroke variables. PVH = periventricular hyperintensities, DWMH = deep white matter hyperintensities, N/A = relationship not tested.
(DOCX)

S6 Table Subtests taken from each language battery. Subtests from the Western Aphasia Battery–Revised (WAB-R), Boston Diagnostic Aphasia Examination (BDAE), and an in-house lexical battery (LB) were chosen to reflect auditory comprehension, naming, and verbal expression skills.
(DOCX)

S7 Table Relationships between demographic and stroke variables. Spearman correlations were used to determine relationships between age and continuous or ordinal variables. Wilcoxon rank sum tests were used to determine one categorical variable and one continuous or ordinal variable. Chi-square tests were used to determine if the binary hypoperfusion variable or history of stroke varied by sex. *Stat*. = the test statistic for the corresponding tests. P-values adjusted for the False Discovery Rate (FDR) are reported as Q-values. * denotes significance at *P/Q* < 0.05, ** denotes significance at *P/Q* < 0.01.
(DOCX)

S8 Table Language data from three patients with severe language deficits. Results from additional behavioral tasks are also included where relevant. (A) Patient 1 received the Western Aphasia Battery–Revised (WAB-R) and additional tests for word span, digit span, and comprehending movement-derived sentences. This patient was non-verbal, and used a communication board. (B) Patient 2 received the WAB-R, the Apraxia Battery for Adults, and additional tests for digit span, word span, and thematic role assignment. (C) Patient 3 received the Boston Diagnostic Aphasia Examination (BDAE), which included a rating scale for profiling speech characteristics. Additional tests included Pyramids & Palm Trees as well as Kissing and Dancing.
(DOCX)
