# Supplementary material for: The contribution of white matter pathology, hypoperfusion, lesion load, and stroke recurrence to language deficits following acute subcortical left hemisphere stroke
Source: PLoS One. 2022 Oct 26;17(10):e0275664. doi: 10.1371/journal.pone.0275664 (PMC9604977; doi:10.1371/journal.pone.0275664)
Supplement: S1 Table — Electronic medical records of all participants were canvassed for a significant history of atrial fibrillation (AF), coronary artery disease (CAD), diabetes mellitus (DM), hyperlipidemia (HL), hypertension (HTN), tobacco/smoking (TOB), and substance abuse (SA). Years of education, handedness, stroke etiology, and NIH stroke scale score on admission (NIHSS) were recorded when present. SVID: Small vessel ischemic disease. “X” denotes fields where no information was found in patient records. (DOCX) [file pone.0275664.s001.docx]

| **Pt #** | **Sex** | **Age** | **Handedness** | **Yrs Education** | **Stroke Etiology** | **NIHSS** | **Prior Stroke?** | **Comorbidities** |
| --- | --- | --- | --- | --- | --- | --- | --- | --- |
| 1 | F | 36 | R | 14 | drug use | 2 | No | DM, HL, HTN, SA |
| 2 | M | 46 | R | 12 | SVID or cardioembolic | 3 | Yes | HL, HTN, SA, TOB |
| 3 | F | 72 | R | 20 | SVID | 4 | Yes | HL, HTN, TOB |
| 4 | M | 27 | X | 16 | thrombotic | X | No | None |
| 5 | F | 15 | X | X | X | X | No | X |
| 6 | M | 62 | R | X | SVID | X | No | AF, CAD, HL, HTN |
| 7 | F | 62 | X | X | X | X | Yes | CAD, DN, HL, HTN, TOB |
| 8 | F | 73 | X | X | thrombotic | X | No | HTN, CAD |
| 9 | M | 44 | X | X | SVID | 5 | No | HTN |
| 10 | F | 20 | L | 12 | embolic | X | No | None |
| 11 | F | 31 | R | X | SVID | 7 | Yes | HL, HTN |
| 12 | M | 52 | R | 12 | SVID | 2 | No | DM, SA |
| 13 | M | 69 | X | X | X | X | Yes | X |
| 14 | M | 46 | R | X | SVID | X | No | HTN, TOB |
| 15 | F | 29 | R | 11 | SVID | X | No | HTN |
| 16 | M | 53 | R | 12 | X | X | No | HL, SA |
| 17 | M | 48 | R | 15.5 | SVID | 1 | No | None |
| 18 | M | 37 | X | X | cerebral vasculitis | X | No | SA |
| 19 | M | 32 | R | X | thrombotic | X | No | HTN |
| 20 | M | 60 | R | 13 | thrombotic | 1 | No | DM, HL, HTN |
| 21 | F | 50 | X | X | X | X | Yes | X |
| 22 | F | 75 | R | X | SVID | X | Yes | HL, HTN, TOB |
| 23 | F | 60 | X | 12 | X | X | No | DM, HL, HTN, TOB |
| 24 | F | 57 | X | X | SVID | X | No | HTN |
| 25 | M | 59 | X | 12 | thrombotic | X | No | HTN |
| 26 | M | 61 | R | 15 | thrombotic | 1 | No | HL, HTN |
| 27 | F | 67 | R | X | X | X | Yes | HL, HTN |
| 28 | F | 50 | X | X | SVID | X | No | HTN, SA |
| 29 | F | 56 | X | X | X | X | No | DM, HTN |
| 30 | M | 63 | R | 16 | X | X | No | HL |
| 31 | M | 56 | L | 16 | thrombotic | 24 | Yes | CAD, DM, HL, HTN |
| 32 | M | 68 | R | X | X | X | No | DM, HTN, TOB |
| 33 | M | 70 | R | X | SVID | 14 | No | DM, HL, HTN, TOB |
| 34 | M | 48 | R | X | X | 1 | No | None |
| 35 | M | 49 | R | X | thrombotic | X | No | HTN |
| 36 | M | 58 | R | X | SVID | X | No | HTN |
| 37 | F | 84 | X | X | SVID | X | No | AF, DM, HL, HTN |
| 38 | M | 76 | R | X | thrombotic | X | No | None |
| 39 | M | 60 | R | X | thrombotic | 2 | No | HTN, TOB |
| 40 | F | 45 | R | X | SVID | X | No | SA, TOB |

| **Pt #** | **Sex** | **Age** | **Handedness** | **Yrs Education** | **Stroke Etiology** | **NIHSS** | **Prior Stroke?** | **Comorbidities** |
| --- | --- | --- | --- | --- | --- | --- | --- | --- |
| 41 | M | 59 | X | X | SVID | X | Yes | HL, HTN |
| 42 | F | 66 | R | X | thrombotic | 1 | Yes | DM, HL |
| 43 | M | 64 | R | 16 | SVID | 7 | Yes | HL, HTN |
| 44 | M | 48 | R | 11 | SVID | 5 | Yes | DM, HTN |
| 45 | F | 34 | X | X | X | X | No | X |
| 46 | F | 48 | R | 10 | thrombotic | 2 | Yes | HTN, SA |
| 47 | M | 66 | R | X | SVID | X | No | HL, HTN |
| 48 | M | 76 | R | 4 | SVID | X | No | DM, HTN |
| 49 | M | 60 | R | X | SVID | 1 | No | DM |
| 50 | M | 70 | L | 14 | SVID | 3 | No | DM, HL, HTN |
| 51 | M | 58 | L | 12 | thrombotic | X | No | HL, HTN |
| 52 | M | 71 | R | X | SVID | X | Yes | TOB |
| 53 | M | 65 | X | X | SVID | X | No | TOB |
| 54 | F | 70 | R | 10 | SVID | 2 | Yes | DM, HL, HTN |
| 55 | F | 56 | R | 12 | thrombotic | 3 | Yes | DM, HL, HTN, TOB |
| 56 | F | 77 | R | X | SVID | 2 | Yes | DM, HL, HTN |
| 57 | F | 35 | R | 12 | thrombotic | X | No | TOB |
| 58 | F | 41 | L | X | SVID | X | No | DM, HTN |
| 59 | F | 35 | R | X | embolic | 0 | No | None |
| 60 | F | 67 | X | X | SVID | X | Yes | HTN |
| 61 | M | 54 | R | X | thrombotic | X | Yes | DM, HL |
| 62 | F | 52 | R | X | thrombotic | 3 | No | HTN |
| 63 | F | 76 | R | X | SVID | X | Yes | CAD, DM, HTN |
| 64 | M | 47 | R | 16 | SVID | 6 | No | DM, HL, HTN |
| 65 | F | 51 | X | X | X | X | Yes | X |
| 66 | F | 28 | R | 12 | thrombotic | X | No | HTN |
| 67 | M | 51 | X | X | cryptogenic | X | Yes | DM, HL, HTN |
| 68 | M | 49 | R | 12 | SVID | 4 | No | DM, HL, HTN, SA, TOB |
| 69 | M | 80 | R | X | SVID or thrombotic | 2 | Yes | DM, HTN |
| 70 | M | 53 | R | 11 | SVID | 4 | No | HTN, TOB |
| 71 | F | 56 | R | 18 | SVID | 5 | No | HTN |
| 72 | F | 59 | R | X | thrombotic | 8 | Yes | DM, HTN, TOB |
| 73 | F | 65 | R | X | SVID | X | No | HTN, TOB |
| 74 | M | 58 | X | X | thrombotic | 10 | Yes | HTN, SA |
| 75 | F | 57 | X | X | SVID | X | No | DM, HTN |
| 76 | M | 68 | R | X | SVID | 7 | Yes | HTN |
| 77 | M | 57 | X | X | embolic | 0 | Yes | DM, HTN |
| 78 | F | 79 | X | X | X | X | No | X |
| 79 | M | 61 | X | X | SVID | X | No | HTN, SA |
| 80 | M | 60 | R | 16 | SVID or embolic | 5 | No | HTN, TOB |

**S1 Table. Characteristics of retrospective sample.** Electronic medical records of all participants were canvassed for a significant history of atrial fibrillation (AF), coronary artery disease (CAD), diabetes mellitus (DM), hyperlipidemia (HL), hypertension (HTN), tobacco/smoking (TOB), and substance abuse (SA). Years of education, handedness, stroke etiology, and NIH stroke scale score on admission (NIHSS) were recorded when present. *SVID: small vessel ischemic disease*. “X” denotes fields where no information was found in patient records.
