# Supplementary material for: The contribution of white matter pathology, hypoperfusion, lesion load, and stroke recurrence to language deficits following acute subcortical left hemisphere stroke
Source: PLoS One. 2022 Oct 26;17(10):e0275664. doi: 10.1371/journal.pone.0275664 (PMC9604977; doi:10.1371/journal.pone.0275664)
Supplement: S2 Table — FOV = field of view; TR = repetition time; TE = echo time. (DOCX) [file pone.0275664.s002.docx]

| **Pt #** | **Field Strength (T)** | **FOV** | **TR (ms)** | **TE (ms)** | **Voxel Dimensions (mm)** | **Slice Thickness (mm)** | **Flip Angle (˚)** |
| --- | --- | --- | --- | --- | --- | --- | --- |
| 1 | 3 | 256 * 256 | 12000 | 84 | 0.8984 x 0.8984 x 3.5055 | 3.5 | 90 |
| 2 | 3 | 192 * 192 | 9000 | 98 | 1.1979 x 1.1979 x 3.9863 | 4 | 90 |
| 3 | 3 | 192 * 192 | 12000 | 84 | 1.1979 x 1.1979 x 3.9796 | 5 | 90 |
| 4 | 3 | 256 * 256 | 12000 | 84 | 0.9375 x 0.9375 x 4.9981 | 5 | 90 |
| 5 | 3 | 256 * 256 | 9000 | 98 | 0.9375 x 0.9375 x 5 | 3.5 | 90 |
| 6 | 3 | 256 * 256 | 12000 | 84 | 0.9375 x 0.9375 x 4.7854 | 3.5 | 90 |
| 7 | 3 | 256 * 256 | 9980 | 129 | 0.9375 x 0.9375 x 4.9996 | 3.5 | 90 |
| 8 | 3 | 256 * 256 | 12000 | 84 | 0.9375 x 0.9375 x 4.8000 | 3.5 | 90 |
| 9 | 1.5 | 256 * 256 | 9000 | 100 | 0.9766 x 0.9766 x 5.7375 | 5 | 90 |
| 10 | 3 | 256 * 256 | 5000 | 78 | 0.8984 x 0.8984 x 3.9788 | 4 | 90 |
| 11 | 3 | 128 * 128 | 6800 | 85 | 1.7188 x 1.7188 x 3.6644 | 3 | 90 |
| 12 | 1.5 | 192 * 192 | 3800 | 89 | 1.1979 x 1.1979 x 6.4424 | 5 | 90 |
| 13 | 1.5 | 256 * 256 | 9000 | 113 | 0.9375 x 0.9375 x 5 | 5 | 90 |
| 14 | 1.5 | 256 * 256 | 9980 | 129 | 0.9375 x 0.9375 x 4.9354 | 5 | 90 |
| 15 | 1.5 | 192 * 192 | 6500 | 115 | 1.1979 x 1.1979 x 4.9953 | 5 | 90 |
| 16 | 1.5 | 256 * 256 | 10000 | 80 | 0.9375 x 0.9375 x 4.9663 | 5 | 90 |
| 17 | 3 | 160 * 160 | 5700 | 87 | 1.4375 x 1.4375 x 3.9570 | 4 | 90 |
| 18 | 1.5 | 256 * 256 | 9980 | 119 | 0.9375 x 0.9375 x 4.9989 | 5 | 90 |
| 19 | 1.5 | 256 * 256 | 9980 | 120 | 0.9375 x 0.9375 x 4.9921 | 5 | 90 |
| 20 | 1.5 | 384 * 384 | 7500 | 94 | 0.5990 x 0.5990 x 6.3424 | 5 | 90 |
| 21 | 1.5 | 256 * 256 | 9980 | 117 | 0.9375 x 0.9375 x 5 | 5 | 90 |
| 22 | 3 | 160 * 160 | 5700 | 87 | 1.3750 x 1.3750 x 3.9972 | 4 | 90 |
| 23 | 1.5 | 384 * 384 | 3700 | 89 | 0.5990 x 0.5990 x 5.9994 | 5 | 90 |
| 24 | 1.5 | 256 * 256 | 9000 | 100 | 0.8984 x 0.8984 x 6.8660 | 5 | 90 |
| 25 | 1.5 | 192 * 192 | 6500 | 115 | 1.1979 x 1.1979 x 4.9887 | 5 | 90 |
| 26 | 1.5 | 384 * 384 | 4500 | 96 | 0.5990 x 0.5990 x 5.8613 | 5 | 90 |
| 27 | 1.5 | 256 * 256 | 9980 | 90 | 0.9375 x 0.9375 x 4.9955 | 5 | 90 |
| 28 | 1.5 | 256 * 256 | 9980 | 124 | 0.9375 x 0.9375 x 4.9585 | 5 | 90 |
| 29 | 1.5 | 256 * 256 | 5000 | 80 | 0.9375 x 0.9375 x 4.9000 | 5 | 90 |
| 30 | 1.5 | 256 * 256 | 9980 | 114 | 0.9375 x 0.9375 x 4.5000 | 5 | 90 |
| 31 | 3 | 168 * 168 | 6500 | 93 | 1.4881 x 1.4881 x 3.7304 | 4 | 90 |
| 32 | 1.5 | 256 * 256 | 9980 | 113 | 0.9375 x 0.9375 x 5 | 5 | 90 |
| 33 | 1.5 | 192 * 192 | 5500 | 106 | 1.6198 x 1.6198 x 4.7919 | 5 | 90 |
| 34 | 1.5 | 384 * 384 | 7900 | 94 | 0.5990 x 0.5990 x 6.4350 | 5 | 90 |
| 35 | 1.5 | 256 * 256 | 9980 | 117 | 0.9375 x 0.9375 x 5 | 5 | 90 |
| 36 | 1.5 | 192 * 192 | 6100 | 89 | 1.1979 x 1.1979 x 4.9910 | 5 | 90 |
| 37 | 1.5 | 256 * 256 | 9000 | 100 | 0.8984 x 0.8984 x 4.3281 | 5 | 90 |
| 38 | 1.5 | 256 * 256 | 9000 | 88 | 0.9375 x 0.9375 x 4.9000 | 5 | 90 |
| 39 | 3 | 192 * 192 | 9000 | 98 | 1.1979 x 1.1979 x 3.9552 | 4 | 90 |
| 40 | 1.5 | 384 * 384 | 3700 | 89 | 0.5990 x 0.5990 x 5.9582 | 5 | 90 |

| **Pt #** | **Field Strength (T)** | **FOV** | **TR (ms)** | **TE (ms)** | **Voxel Dimensions (mm)** | **Slice Thickness (mm)** | **Flip Angle (˚)** |
| --- | --- | --- | --- | --- | --- | --- | --- |
| 41 | 1.5 | 256 * 256 | 9980 | 104 | 0.9375 x 0.9375 x 4.9990 | 5 | 90 |
| 42 | 1.5 | 192* 192 | 6100 | 89 | 1.1979 x 1.1979 x 4.9954 | 5 | 90 |
| 43 | 3 | 192* 192 | 9000 | 98 | 1.1979 x 1.1979 x 3.9090 | 4 | 90 |
| 44 | 1.5 | 160* 160 | 9307 | 85 | 1.3750 x 1.3750 x 4.7294 | 5 | 90 |
| 45 | 1.5 | 256 * 256 | 9980 | 123 | 0.9375 x 0.9375 x 4.9675 | 5 | 90 |
| 46 | 1.5 | 256 * 256 | 10000 | 85 | 0.9375 x 0.9375 x 4.9282 | 5 | 90 |
| 47 | 1.5 | 192* 192 | 5100 | 89 | 1.1979 x 1.1979 x 4.9832 | 5 | 90 |
| 48 | 3 | 256 * 256 | 5000 | 78 | 0.8984 x 0.8984 x 3.6908 | 4 | 90 |
| 49 | 3 | 192* 192 | 9316 | 98 | 1.1979 x 1.1979 x 3.9723 | 4 | 90 |
| 50 | 3 | 192 * 192 | 9000 | 98 | 1.1979 x 1.1979 x 3.8880 | 4 | 90 |
| 51 | 1.5 | 384* 384 | 7500 | 94 | 0.5990 x 0.5990 x 6.4719 | 5 | 90 |
| 52 | 1.5 | 256 * 256 | 9000 | 100 | 0.8984 x 0.8984 x 6.6663 | 5 | 90 |
| 53 | 1.5 | 256 * 256 | 9980 | 121 | 0.9375 x 0.9375 x 4.9931 | 5 | 90 |
| 54 | 1.5 | 384 * 384 | 7500 | 94 | 0.5990 x 0.5990 x 6.4109 | 5 | 90 |
| 55 | 1.5 | 384 * 384 | 7500 | 94 | 0.5990 x 0.5990 x 6.4650 | 5 | 90 |
| 56 | 3 | 256 * 256 | 8800 | 84 | 0.8984 x 0.8984 x 3.8226 | 4 | 90 |
| 57 | 1.5 | 256 * 256 | 9980 | 117 | 0.9375 x 0.9375 x 5.0000 | 5 | 90 |
| 58 | 1.5 | 256 * 256 | 9980 | 125 | 0.9375 x 0.9375 x 4.9914 | 5 | 90 |
| 59 | 3 | 192 * 192 | 9800 | 98 | 1.1979 x 1.1979 x 3.9685 | 4 | 90 |
| 60 | 1.5 | 256 * 256 | 10000 | 131 | 0.9375 x 0.9375 x 4.9377 | 5 | 90 |
| 61 | 1.5 | 192 * 192 | 3800 | 89 | 1.1979 x 1.1979 x 6.4679 | 5 | 90 |
| 62 | 3 | 192 * 192 | 8900 | 98 | 1.1979 x 1.1979 x 3.9338 | 4 | 90 |
| 63 | 1.5 | 192 * 192 | 6400 | 114 | 1.1979 x 1.1979 x 4.7714 | 5 | 90 |
| 64 | 3 | 192 * 192 | 9000 | 98 | 1.1979 x 1.1979 x 3.7167 | 4 | 90 |
| 65 | 1.5 | 256 * 256 | 6400 | 114 | 0.9375 x 0.9375 x 5 | 5 | 90 |
| 66 | 1.5 | 384 * 384 | 7500 | 94 | 0.5990 x 0.5990 x 6.3883 | 5 | 90 |
| 67 | 1.5 | 256 * 256 | 9000 | 100 | 0.8984 x 0.8984 x 4.0224 | 5 | 90 |
| 68 | 3 | 192 * 192 | 9000 | 98 | 1.1979 x 1.1979 x 3.8761 | 4 | 90 |
| 69 | 3 | 192 * 192 | 9000 | 98 | 1.1979 x 1.1979 x 3.8923 | 4 | 90 |
| 70 | 3 | 256 * 256 | 9400 | 94 | 0.8984 x 0.8984 x 3.6603 | 3.5 | 90 |
| 71 | 3 | 256 * 256 | 8800 | 84 | 0.8984 x 0.8984 x 3.3708 | 3.5 | 90 |
| 72 | 1.5 | 384 * 384 | 7500 | 94 | 0.5990 x 0.5990 x 6.4867 | 5 | 90 |
| 73 | 1.5 | 256 * 256 | 9980 | 127 | 0.9375 x 0.9375 x 4.9804 | 5 | 90 |
| 74 | 1.5 | 160 * 160 | 8500 | 85 | 1.3750 x 1.3750 x 4.9411 | 5 | 90 |
| 75 | 1.5 | 192 * 192 | 6100 | 89 | 1.1979 x 1.1979 x 4.9573 | 5 | 90 |
| 76 | 3 | 256 * 256 | 10000 | 84 | 0.8984 x 0.8984 x 3.5066 | 3.5 | 90 |
| 77 | 1.5 | 192 * 192 | 3800 | 89 | 1.1979 x 1.1979 x 6.2871 | 5 | 90 |
| 78 | 1.5 | 256 * 256 | 6400 | 114 | 0.9375 x 0.9375 x 4.7000 | 5 | 90 |
| 79 | 1.5 | 256 * 256 | 9980 | 130 | 0.9375 x 0.9375 x 4.8633 | 5 | 90 |
| 80 | 3 | 192 * 192 | 9316 | 98 | 1.1979 x 1.1979 x 3.9563 | 4 | 90 |

**S2 Table. Diffusion-weighted imaging parameters.** *FOV* = field of view; *TR* = repetition time; *TE =* echo time.
