# Supplementary material for: The contribution of white matter pathology, hypoperfusion, lesion load, and stroke recurrence to language deficits following acute subcortical left hemisphere stroke
Source: PLoS One. 2022 Oct 26;17(10):e0275664. doi: 10.1371/journal.pone.0275664 (PMC9604977; doi:10.1371/journal.pone.0275664)
Supplement: S3 Table — FOV = field of view; TR = repetition time; TE = echo time. (DOCX) [file pone.0275664.s003.docx]

| **Pt #** | **Field Strength (T)** | **FOV** | **TR (ms)** | **TE (ms)** | **Voxel Dimensions (mm)** | **Slice Thickness (mm)** | **Flip Angle (˚)** |
| --- | --- | --- | --- | --- | --- | --- | --- |
| 1 | 3 | 384 * 384 | 4000 | 99 | 0.5729 x 0.5729 x 3.8155 | 4 | 152 |
| 2 | 3 | 640 * 480 | 4190 | 96 | 0.3594 x 0.3594 x 4.9828 | 4 | 160 |
| 3 | 3 | 640 * 480 | 3940 | 96 | 0.3594 x 0.3594 x 4.9745 | 4 | 160 |
| 4 | 1.5 | 512 * 512 | 4400 | 104 | 0.4687 x 0.4687 x 4.9981 | 5 | 90 |
| 5 | 1.5 | 512 * 512 | 4350 | 107 | 0.4688 x 0.4688 x 5 | 5 | 90 |
| 6 | 1.5 | 512 * 512 | 4000 | 107 | 0.4688 x 0.4688 x 4.7854 | 5 | 90 |
| 7 | 1.5 | 512 * 512 | 4300 | 96 | 0.4688 x 0.4688 x 5 | 5 | 90 |
| 8 | 1.5 | 512 * 512 | 4500 | 102 | 0.4688 x 0.4688 x 4.8000 | 5 | 90 |
| 9 | 1.5 | 512 * 512 | 3180 | 105 | 0.5078 x 0.5078 x 7.3896 | 5 | 180 |
| 10 | 1.5 | 512 * 512 | 4190 | 120 | 0.4492 x 0.4492 x 4.9635 | 5 | 90 |
| 11 | 3 | 384 * 324 | 4000 | 97 | 0.5729 x 0.5729 x 3.9351 | 4 | 121 |
| 12 | 1.5 | 320 * 244 | 3800 | 93 | 0.7188 x 0.7188 x 3.9452 | 5 | 90 |
| 13 | 1.5 | 512 * 512 | 4500 | 96 | 0.4687 x 0.4688 x 4.8000 | 5 | 90 |
| 14 | 1.5 | 512 * 512 | 4000 | 105 | 0.4688 x 0.4687 x 4.9354 | 5 | 90 |
| 15 | 1.5 | 512 * 384 | 3600 | 100 | 0.4492 x 0.4492 x 4.9778 | 5 | 180 |
| 16 | 1.5 | 512 * 512 | 3580 | 105 | 0.4492 x 0.4492 x 4.9663 | 5 | 90 |
| 17 | 3 | 320 * 272 | 4100 | 95 | 0.7188 x 0.7188 x 3.9942 | 4 | 90 |
| 18 | 1.5 | 512 * 512 | 4080 | 107 | 0.4688 x 0.4688 x 4.9989 | 5 | 90 |
| 19 | 1.5 | 512 * 512 | 4400 | 107 | 0.4688 x 0.4688 x 4.9973 | 5 | 90 |
| 20 | 1.5 | 256 * 256 | 5940 | 117 | 0.9766 x 0.9766 x 3.9030 | 4 | 156 |
| 21 | 1.5 | 512 * 512 | 4450 | 96 | 0.4687 x 0.4688 x 4.9000 | 5 | 90 |
| 22 | 3 | 320 * 272 | 4500 | 95 | 0.6875 x 0.6875 x 3.9972 | 4 | 90 |
| 23 | 1.5 | 512 * 384 | 4620 | 98 | 0.4492 x 0.4492 x 6.4998 | 5 | 150 |
| 24 | 1.5 | 512 * 512 | 3180 | 105 | 0.5078 x 0.5078 x 7.4168 | 5 | 180 |
| 25 | 1.5 | 512 * 464 | 3600 | 100 | 0.4492 x 0.4492 x 4.9887 | 5 | 180 |
| 26 | 1.5 | 320 * 240 | 4500 | 112 | 0.7188 x 0.7188 x 4.8708 | 5 | 147 |
| 27 | 1.5 | 512 * 512 | 4200 | 107 | 0.4687 x 0.4688 x 4.9955 | 5 | 90 |
| 28 | 1.5 | 512 * 512 | 4200 | 107 | 0.4687 x 0.4688 x 4.9585 | 5 | 90 |
| 29 | 1.5 | 512 * 512 | 4400 | 107 | 0.4687 x 0.4688 x 4.9000 | 5 | 90 |
| 30 | 1.5 | 512 * 512 | 5600 | 102 | 0.4687 x 0.4688 x 4.5000 | 5 | 90 |
| 31 | 1.5 | 320 * 256 | 4600 | 98 | 0.7188 x 0.7188 x 4.8970 | 5 | 127 |
| 32 | 1.5 | 512 * 512 | 4350 | 107 | 0.4688 x 0.4688 x 5 | 5 | 90 |
| 33 | 1.5 | 256 * 192 | 4580 | 100 | 0.9766 x 0.9766 x 4.7919 | 5 | 150 |
| 34 | 1.5 | 384 * 324 | 4510 | 105 | 0.5729 x 0.5729 x 3.9600 | 4 | 150 |
| 35 | 1.5 | 512 * 512 | 4350 | 96 | 0.4688 x 0.4688 x 5 | 5 | 90 |
| 36 | 1.5 | 320 * 256 | 6090 | 96 | 0.7188 x 0.7188 x 4.9910 | 5 | 150 |
| 37 | 1.5 | 512 * 512 | 3180 | 105 | 0.5078 x 0.5078 x 7.4339 | 5 | 180 |
| 38 | 1.5 | 512 * 512 | 4000 | 99 | 0.4688 x 0.4688 x 4.9000 | 5 | 90 |
| 39 | 3 | 640 * 480 | 4193 | 96 | 0.3594 x 0.3594 x 4.9440 | 4 | 160 |
| 40 | 1.5 | 512 * 384 | 4620 | 98 | 0.4492 x 0.4492 x 5.8767 | 4 | 150 |

| **Pt #** | **Field Strength (T)** | **FOV** | **TR (ms)** | **TE (ms)** | **Voxel Dimensions (mm)** | **Slice Thickness (mm)** | **Flip Angle (˚)** |
| --- | --- | --- | --- | --- | --- | --- | --- |
| 41 | 1.5 | 512 * 512 | 3950 | 106 | 0.4688 x 0.4688 x 4.9990 | 5 | 90 |
| 42 | 3 | 512 * 512 | 1861 | 100 | 0.4492 x 0.4492 x 4.9441 | 5 | 90 |
| 43 | 3 | 192 * 192 | 9000 | 98 | 0.5729 x 0.5729 x 3.9600 | 4 | 90 |
| 44 | 1.5 | 320 * 270 | 4000 | 100 | 0.6875 x 0.6875 x 3.7835 | 4 | 150 |
| 45 | 1.5 | 512 * 512 | 4500 | 102 | 0.4687 x 0.4688 x 4.9675 | 5 | 90 |
| 46 | 1.5 | 512 * 512 | 3900 | 98 | 0.4688 x 0.4688 x 4.9283 | 5 | 90 |
| 47 | 1.5 | 320 * 272 | 4010 | 98 | 0.7188 x 0.7188 x 4.9832 | 5 | 139 |
| 48 | 3 | 320 * 272 | 4630 | 119 | 0.6875 x 0.6875 x 4.7981 | 4 | 90 |
| 49 | 3 | 640 * 500 | 3940 | 96 | 0.3594 x 0.3594 x 4.9654 | 4 | 160 |
| 50 | 1.5 | 320 * 250 | 9000 | 101 | 0.7188 x 0.7188 x 3.9531 | 4 | 150 |
| 51 | 1.5 | 384 * 288 | 5530 | 107 | 0.5990 x 0.5990 x 3.9827 | 4 | 150 |
| 52 | 1.5 | 512 * 512 | 3180 | 105 | 0.5078 x 0.5078 x 4.4851 | 5 | 180 |
| 53 | 1.5 | 512 * 512 | 4083 | 107 | 0.4687 x 0.4688 x 4.9932 | 5 | 90 |
| 54 | 1.5 | 384 * 288 | 4510 | 105 | 0.5990 x 0.5990 x 3.9540 | 4 | 150 |
| 55 | 1.5 | 384 * 288 | 5530 | 107 | 0.5990 x 0.5990 x 3.9354 | 4 | 150 |
| 56 | 3 | 384 * 312 | 5000 | 99 | 0.5990 x 0.5990 x 3.8226 | 4 | 146 |
| 57 | 1.5 | 384 * 288 | 5530 | 107 | 0.5990 x 0.5990 x 3.9569 | 4 | 150 |
| 58 | 1.5 | 512 * 512 | 4000 | 104 | 0.4688 x 0.4688 x 4.9914 | 5 | 90 |
| 59 | 3 | 640 * 480 | 4200 | 96 | 0.3594 x 0.3594 x 4.9439 | 4 | 160 |
| 60 | 1.5 | 512 * 512 | 3000 | 103 | 0.4688 x 0.4687 x 4.9377 | 5 | 90 |
| 61 | 1.5 | 320 * 320 | 3550 | 98 | 0.7188 x 0.7188 x 4.9753 | 5 | 129 |
| 62 | 3 | 320 * 250 | 4050 | 97 | 0.6875 x 0.6875 x 3.9338 | 4 | 120 |
| 63 | 1.5 | 512 * 384 | 4150 | 100 | 0.4492 x 0.4492 x 4.7995 | 5 | 150 |
| 64 | 3 | 384 * 348 | 4050 | 102 | 0.5729 x 0.5729 x 3.3788 | 4 | 120 |
| 65 | 1.5 | 512 * 512 | 4217 | 96 | 0.4688 x 0.4688 x 5 | 5 | 90 |
| 66 | 1.5 | 384 * 288 | 5530 | 107 | 0.5990 x 0.5990 x 3.9313 | 4 | 150 |
| 67 | 1.5 | 512 * 512 | 3180 | 105 | 0.4883 x 0.4883 x 3.4589 | 5 | 180 |
| 68 | 3 | 384 * 300 | 4050 | 102 | 0.5729 x 0.5729 x 3.8761 | 4 | 120 |
| 69 | 3 | 640 * 480 | 3940 | 96 | 0.3594 x 0.3594 x 4.8653 | 4 | 160 |
| 70 | 3 | 384 * 324 | 4000 | 97 | 0.5729 x 0.5729 x 3.9840 | 4 | 140 |
| 71 | 3 | 384 * 324 | 4000 | 97 | 0.5729 x 0.5729 x 3.6690 | 4 | 140 |
| 72 | 1.5 | 256 * 256 | 5940 | 117 | 0.8594 x 0.8594 x 3.9918 | 4 | 120 |
| 73 | 1.5 | 512 * 512 | 4000 | 103 | 0.4687 x 0.4688 x 4.9804 | 5 | 90 |
| 74 | 1.5 | 384 * 300 | 5530 | 107 | 0.5990 x 0.5990 x 3.9303 | 4 | 150 |
| 75 | 1.5 | 256 * 232 | 4850 | 95 | 0.8594 x 0.8594 x 2.4787 | 2.5 | 144 |
| 76 | 3 | 384 * 324 | 4000 | 97 | 0.5729 x 0.5729 x 3.8167 | 4 | 140 |
| 77 | 1.5 | 320 * 240 | 4260 | 93 | 0.7188 x 0.7188 x 4.9285 | 5 | 150 |
| 78 | 1.5 | 512 * 512 | 4167 | 108 | 0.4688 x 0.4688 x 4.7000 | 5 | 90 |
| 79 | 1.5 | 512 * 512 | 4500 | 103 | 0.4687 x 0.4688 x 4.8607 | 5 | 90 |
| 80 | 3 | 640 * 520 | 4460 | 96 | 0.3750 x 0.3750 x 4.9453 | 4 | 160 |

**S3 Table. T2-weighted imaging parameters.** *FOV* = field of view; *TR* = repetition time; *TE =* echo time.
