# Supplementary material for: The contribution of white matter pathology, hypoperfusion, lesion load, and stroke recurrence to language deficits following acute subcortical left hemisphere stroke
Source: PLoS One. 2022 Oct 26;17(10):e0275664. doi: 10.1371/journal.pone.0275664 (PMC9604977; doi:10.1371/journal.pone.0275664)
Supplement: S4 Table — FOV = field of view; TR = repetition time; TE = echo time. (DOCX) [file pone.0275664.s004.docx]

| **Pt #** | **Field Strength (T)** | **FOV** | **TR (ms)** | **TE (ms)** | **Voxel Dimensions (mm)** | **Slice Thickness (mm)** | **Flip Angle (˚)** |
| --- | --- | --- | --- | --- | --- | --- | --- |
| 1 | 3 | 256 * 200 | 9000 | 123 | 0.8984 x 0.8984 x 3.8155 | 4 | 154 |
| 2 | 3 | 320 * 240 | 9000 | 105 | 0.7188 x 0.7188 x 3.9863 | 4 | 160 |
| 3 | 3 | 320 * 240 | 9000 | 105 | 0.7188 x 0.7188 x 3.9796 | 4 | 157 |
| 4 | 1.5 | 512 * 512 | 8802 | 124 | 0.4687 x 0.4687 x 4.9981 | 5 | 90 |
| 5 | 1.5 | 512 * 512 | 8802 | 137 | 0.4688 x 0.4688 x 5 | 5 | 90 |
| 6 | 1.5 | 512 * 512 | 8802 | 147 | 0.4688 x 0.4688 x 4.7854 | 5 | 90 |
| 7 | 1.5 | 512 * 512 | 8802 | 124 | 0.4687 x 0.4687 x 4.9987 | 5 | 90 |
| 8 | 1.5 | 512 * 512 | 8802 | 133 | 0.4688 x 0.4688 x 4.8000 | 5 | 90 |
| 9 | 1.5 | 256 * 256 | 9000 | 105 | 0.9766 x 0.9766 x 3.9986 | 5 | 90 |
| 10 | 1.5 | 256 * 256 | 6000 | 120 | 0.8984 x 0.8984 x 4.9635 | 5 | 90 |
| 11 | 3 | 256 * 200 | 9000 | 119 | 0.8984 x 0.8984 x 3.9351 | 4 | 154 |
| 12 | 1.5 | 256 * 204 | 9000 | 121 | 0.8984 x 0.8984 x 4.9557 | 5 | 180 |
| 13 | 1.5 | 512 * 512 | 8802 | 133 | 0.4687 x 0.4688 x 5 | 5 | 90 |
| 14 | 1.5 | 512 * 512 | 8802 | 138 | 0.4688 x 0.4687 x 4.9354 | 5 | 90 |
| 15 | 1.5 | 256 * 204 | 9000 | 120 | 0.8984 x 0.8984 x 4.9778 | 5 | 180 |
| 16 | 1.5 | 512 * 512 | 8802 | 124 | 0.4492 x 0.4492 x 4.9663 | 5 | 90 |
| 17 | 3 | 320 * 240 | 9000 | 121 | 0.7188 x 0.7188 x 3.9570 | 4 | 129 |
| 18 | 1.5 | 512 * 512 | 8802 | 137 | 0.4688 x 0.4688 x 4.9989 | 5 | 90 |
| 19 | 1.5 | 512 * 512 | 8802 | 137 | 0.4688 x 0.4688 x 4.9973 | 5 | 90 |
| 20 | 1.5 | 256 * 256 | 8000 | 102 | 0.9766 x 0.9766 x 3.9030 | 4 | 150 |
| 21 | 1.5 | 512 * 512 | 8802 | 133 | 0.4688 x 0.4688 x 5 | 5 | 90 |
| 22 | 3 | 320 * 240 | 9000 | 121 | 0.7188 x 0.7188 x 3.9972 | 4 | 132 |
| 23 | 1.5 | 512 * 448 | 9000 | 90 | 0.4492 x 0.4492 x 4.1998 | 5 | 150 |
| 24 | NO FLAIR | NO FLAIR | NO FLAIR | NO FLAIR | NO FLAIR | NO FLAIR | NO FLAIR |
| 25 | 1.5 | 256 * 232 | 9000 | 120 | 0.8984 x 0.8984 x 4.9887 | 5 | 180 |
| 26 | 1.5 | 256 * 256 | 6300 | 102 | 0.8984 x 0.8984 x 4.9545 | 5 | 180 |
| 27 | 1.5 | 512 * 512 | 8802 | 147 | 0.4687 x 0.4688 x 4.9954 | 5 | 90 |
| 28 | 1.5 | 512 * 512 | 8802 | 133 | 0.4687 x 0.4688 x 4.9585 | 5 | 90 |
| 29 | 1.5 | 512 * 512 | 8802 | 124 | 0.4687 x 0.4688 x 4.9000 | 5 | 90 |
| 30 | 1.5 | 512 * 512 | 8802 | 133 | 0.4687 x 0.4688 x 4.5000 | 5 | 90 |
| 31 | 1.5 | 256 * 216 | 9000 | 117 | 0.8984 x 0.8984 x 4.8970 | 5 | 150 |
| 32 | 1.5 | 512 * 512 | 8802 | 137 | 0.4688 x 0.4688 x 5 | 5 | 90 |
| 33 | 1.5 | 256 * 204 | 9000 | 107 | 0.9766 x 0.9766 x 4.7919 | 5 | 180 |
| 34 | 1.5 | 320 * 290 | 9000 | 101 | 0.6875 x 0.6875 x 3.9600 | 4 | 150 |
| 35 | 1.5 | 512 * 512 | 8802 | 137 | 0.4688 x 0.4688 x 5 | 5 | 90 |
| 36 | 1.5 | 256 * 204 | 7000 | 111 | 0.8984 x 0.8984 x 4.9910 | 5 | 180 |
| 37 | 1.5 | 256 * 256 | 32 | 6 | 0.9766 x 0.9766 x 0.9994 | 1 | 25 |
| 38 | 1.5 | 512 * 512 | 8802 | 104 | 0.4688 x 0.4688 x 4.9000 | 5 | 90 |
| 39 | 3 | 256 * 256 | 9000 | 90 | 0.8984 x 0.8984 x 3.9552 | 4 | 150 |
| 40 | 1.5 | 512 * 448 | 9000 | 90 | 0.4492 x 0.4492 x 4.3469 | 5 | 150 |

| **Pt #** | **Field Strength (T)** | **FOV** | **TR (ms)** | **TE (ms)** | **Voxel Dimensions (mm)** | **Slice Thickness (mm)** | **Flip Angle (˚)** |
| --- | --- | --- | --- | --- | --- | --- | --- |
| 41 | 1.5 | 512 * 512 | 8802 | 104 | 0.4688 x 0.4688 x 4.9990 | 5 | 90 |
| 42 | 3 | 512 * 512 | 9000 | 80 | 0.4492 x 0.4492 x 4.9441 | 5 | 90 |
| 43 | 3 | 320 * 240 | 9000 | 105 | 0.7188 x 0.7188 x 3.9090 | 4 | 160 |
| 44 | 1.5 | 320 * 240 | 9000 | 100 | 0.7188 x 0.7188 x 3.7835 | 4 | 154 |
| 45 | 1.5 | 512 * 512 | 8802 | 147 | 0.4687 x 0.4688 x 4.9675 | 5 | 90 |
| 46 | NO FLAIR | NO FLAIR | NO FLAIR | NO FLAIR | NO FLAIR | NO FLAIR | NO FLAIR |
| 47 | 1.5 | 256 * 224 | 8800 | 112 | 0.8594 x 0.8594 x 4.9832 | 5 | 150 |
| 48 | 3 | 256 * 256 | 9000 | 129 | 0.8594 x 0.8594 x 4.7981 | 4 | 130 |
| 49 | 3 | 320 * 260 | 9000 | 106 | 0.7188 x 0.7188 x 3.9723 | 4 | 160 |
| 50 | 1.5 | 320 * 250 | 9000 | 101 | 0.7188 x 0.7188 x 3.9531 | 4 | 150 |
| 51 | 1.5 | 320 * 250 | 9000 | 101 | 0.7188 x 0.7188 x 3.9827 | 4 | 150 |
| 52 | 1.5 | 256 * 256 | 32 | 6 | 0.9766 x 0.9766 x 0.9976 | 1 | 25 |
| 53 | 1.5 | 512 * 512 | 8802 | 147 | 0.4687 x 0.4688 x 4.9931 | 5 | 90 |
| 54 | 1.5 | 320 * 250 | 9000 | 101 | 0.7188 x 0.7188 x 3.9451 | 4 | 150 |
| 55 | 1.5 | 320 * 250 | 9000 | 101 | 0.7188 x 0.7188 x 3.9785 | 4 | 150 |
| 56 | 3 | 256 * 200 | 9000 | 119 | 0.8984 x 0.8984 x 3.8226 | 4 | 154 |
| 57 | 1.5 | 320 * 250 | 9000 | 101 | 0.7188 x 0.7188 x 3.9785 | 5 | 150 |
| 58 | 1.5 | 512 * 512 | 8802 | 125 | 0.4687 x 0.4688 x 4.9914 | 5 | 90 |
| 59 | 3 | 320 * 240 | 9000 | 105 | 0.7188 x 0.7188 x 3.9551 | 4 | 160 |
| 60 | 1.5 | 512 * 512 | 8802 | 138 | 0.4688 x 0.4687 x 4.9377 | 5 | 90 |
| 61 | 1.5 | 256 * 204 | 9000 | 121 | 0.8984 x 0.8984 x 4.9753 | 5 | 180 |
| 62 | 3 | 320 * 240 | 9000 | 105 | 0.7188 x 0.7188 x 3.9338 | 4 | 160 |
| 63 | 1.5 | 256 * 204 | 9000 | 120 | 0.8984 x 0.8984 x 4.8300 | 5 | 180 |
| 64 | 3 | 320 * 320 | 9000 | 105 | 0.7188 x 0.7188 x 3.3788 | 4 | 151 |
| 65 | 1.5 | 512 * 512 | 8802 | 137 | 0.4688 x 0.4688 x 5 | 5 | 90 |
| 66 | 1.5 | 320 * 250 | 9000 | 101 | 0.7188 x 0.7188 x 3.9313 | 4 | 150 |
| 67 | 1.5 | 256 * 256 | 32 | 6 | 1.0156 x 1.0156 x 0.9976 | 1 | 25 |
| 68 | 3 | 320 * 240 | 9000 | 105 | 0.7188 x 0.7188 x 3.8761 | 4 | 160 |
| 69 | 3 | 320 * 230 | 9000 | 105 | 0.7188 x 0.7188 x 3.8923 | 4 | 160 |
| 70 | 3 | 256 * 200 | 9000 | 119 | 0.8984 x 0.8984 x 3.9840 | 4 | 154 |
| 71 | 3 | 256 * 200 | 9000 | 119 | 0.8984 x 0.8984 x 3.6690 | 4 | 154 |
| 72 | 1.5 | 320 * 250 | 9000 | 101 | 0.7188 x 0.7188 x 3.9918 | 4 | 150 |
| 73 | 1.5 | 512 * 512 | 8802 | 138 | 0.4687 x 0.4688 x 4.9804 | 5 | 90 |
| 74 | 1.5 | 320 * 250 | 9000 | 100 | 0.7188 x 0.7188 x 3.9529 | 4 | 154 |
| 75 | 1.5 | 256 * 256 | 5000 | 335 | 1.0156 x 1.0156 x 1.0958 | 3 | 120 |
| 76 | 3 | 256 * 212 | 9000 | 119 | 0.8984 x 0.8984 x 3.8167 | 4 | 154 |
| 77 | 1.5 | 256 * 204 | 9000 | 121 | 0.8984 x 0.8984 x 4.9453 | 5 | 180 |
| 78 | 1.5 | 512 * 512 | 8802 | 133 | 0.4688 x 0.4688 x 4.7000 | 5 | 90 |
| 79 | 1.5 | 512 * 512 | 8802 | 133 | 0.4687 x 0.4688 x 4.8607 | 5 | 90 |
| 80 | 3 | 384 * 324 | 9000 | 107 | 0.6510 x 0.6510 x 3.9563 | 4 | 155 |

**S4 Table. Fluid-attenuated inversion recovery imaging parameters.** *FOV* = field of view; *TR* = repetition time; *TE =* echo time.
