# Supplementary material for: The contribution of white matter pathology, hypoperfusion, lesion load, and stroke recurrence to language deficits following acute subcortical left hemisphere stroke
Source: PLoS One. 2022 Oct 26;17(10):e0275664. doi: 10.1371/journal.pone.0275664 (PMC9604977; doi:10.1371/journal.pone.0275664)
Supplement: S5 Table — The hypoperfusion metric used was the binary variable reflecting presence of hypoperfusion versus no hypoperfusion. Wilcoxon rank sum tests or chi-square tests were used to determine if stroke variables varied by magnet field strength. Spearman correlations were used to determine possible relationships between voxel size and thickness and related stroke variables. PVH = periventricular hyperintensities, DWMH = deep white matter hyperintensities, N/A = relationship not tested. (DOCX) [file pone.0275664.s005.docx]

| Measure | Magnet Field Strength (1.5T or 3T) | DWI Voxel Size (mm^3^) | T2 Voxel Size (mm^3^) | FLAIR Slice Thickness (mm) |
| --- | --- | --- | --- | --- |
| Acute Lesion Volume (mm^3^) | W = 681.0, *P* = 0.639 | r = 0.038, *P* = 0.735 | N/A | N/A |
| Prior Lesion Volume (mm^3^) | W = 97.0, *P* = 0.755 | N/A | r = -0.193, *P* = 0.324 | N/A |
| % Damage Thalamus | W = 708.0, *P* = 0.842 | r = 0.140, *P* = 0.216 | N/A | N/A |
| % Damage External Capsule | W = 690.0, *P* = 0.653 | r = -0.091, *P* = 0.423 | N/A | N/A |
| % Damage Internal Capsule | W = 638.0, *P* = 0.362 | r = 0.068, *P* = 0.546 | N/A | N/A |
| % Damage Corona Radiata | W = 730.0, *P* = 0.988 | r = -0.047, *P* = 0.679 | N/A | N/A |
| % Damage Putamen | W = 726.5, *P* = 0.991 | r = 0.029, *P* = 0.795 | N/A | N/A |
| % Damage Caudate | W = 652.0, *P* = 0.422 | r = 0.107, *P* = 0.343 | N/A | N/A |
| % Damage Globus Pallidus | W = 747.5, *P* = 0.808 | r = 0.102, *P* = 0.368 | N/A | N/A |
| Fazekas PVH | W = 701.0, *P* = 0.780 | N/A | r = 0.007, *P* = 0.953 | N/A |
| Fazekas DWMH | W = 700.0, *P* = 0.770 | N/A | r = -0.092, *P* = 0.420 | N/A |
| Hypoperfusion | χ^2^ = 0.092, *P* = 0.762 | N/A | N/A | W = 682.5, *P* = 0.595 |

**S5 Table. Relationships between MRI parameters and stroke variables.** The hypoperfusion metric used was the binary variable reflecting presence of hypoperfusion versus no hypoperfusion. Wilcoxon rank sum tests or chi-square tests were used to determine if stroke variables varied by magnet field strength. Spearman correlations were used to determine possible relationships between voxel size and thickness and related stroke variables. PVH = periventricular hyperintensities, DWMH = deep white matter hyperintensities, N/A = relationship not tested
