# Supplementary material for: The contribution of white matter pathology, hypoperfusion, lesion load, and stroke recurrence to language deficits following acute subcortical left hemisphere stroke
Source: PLoS One. 2022 Oct 26;17(10):e0275664. doi: 10.1371/journal.pone.0275664 (PMC9604977; doi:10.1371/journal.pone.0275664)
Supplement: S6 Table — Subtests from the Western Aphasia Battery–Revised (WAB-R), Boston Diagnostic Aphasia Examination (BDAE), and an in-house lexical battery (LB) were chosen to reflect auditory comprehension, naming, and verbal expression skills. (DOCX) [file pone.0275664.s006.docx]

| **Western Aphasia Battery - Revised (WAB-R)** | **Boston Diagnostic Aphasia Examination (BDAE)** | **Lexical Battery (LB)** |
| --- | --- | --- |
| Information content | Simple social responses | Oral naming |
| Fluency, grammatical competence | Word comprehension | Tactile naming |
| Yes/no questions | Complex ideational material | Auditory comprehension |
| Auditory word recognition | Automatized sequences |  |
| Sequential commands | Responsive naming |  |
| Object naming |  |  |
| Word fluency |  |  |
| Sentence completion |  |  |
| Responsive speech |  |  |

**S6 Table. Subtests taken from each language battery.** Subtests from the Western Aphasia Battery – Revised (WAB-R), Boston Diagnostic Aphasia Examination (BDAE), and an in-house lexical battery (LB) were chosen to reflect auditory comprehension, naming, and verbal expression skills.
