# Supplementary material for: The contribution of white matter pathology, hypoperfusion, lesion load, and stroke recurrence to language deficits following acute subcortical left hemisphere stroke
Source: PLoS One. 2022 Oct 26;17(10):e0275664. doi: 10.1371/journal.pone.0275664 (PMC9604977; doi:10.1371/journal.pone.0275664)
Supplement: S7 Table — Spearman correlations were used to determine relationships between age and continuous or ordinal variables. Wilcoxon rank sum tests were used to determine one categorical variable and one continuous or ordinal variable. Chi-square tests were used to determine if the binary hypoperfusion variable or history of stroke varied by sex. Stat. = the test statistic for the corresponding tests. P-values adjusted for the False Discovery Rate (FDR) are reported as Q-values. * denotes significance at P/Q < 0.05, ** denotes significance at P/Q < 0.01. (DOCX) [file pone.0275664.s007.docx]

|  | **Age** | | | **Sex** | | |
| --- | --- | --- | --- | --- | --- | --- |
| **Measure** | ***Stat.*** | ***P*** | ***Q*** | ***Stat.*** | ***P*** | ***Q*** |
| Fazekas PVH | 0.553 | << 0.001** | < 0.001** | 849.5 | 0.565 | 0.672 |
| Fazekas DWMH | 0.534 | << 0.001** | < 0.001** | 839.5 | 0.633 | 0.672 |
| Acute lesion volume | -0.202 | 0.072 | 0.251 | 708.0 | 0.419 | 0.672 |
| Total lesion volume | -0.158 | 0.162 | 0.379 | 687.0 | 0.312 | 0.672 |
| % damage thalamus | -0.170 | 0.131 | 0.367 | 883.5 | 0.373 | 0.672 |
| % damage external capsule | -0.043 | 0.704 | 0.821 | 696.0 | 0.273 | 0.672 |
| % damage internal capsule | 0.009 | 0.937 | 0.937 | 617.5 | 0.089 | 0.672 |
| % damage corona radiata | -0.024 | 0.833 | 0.897 | 714.0 | 0.444 | 0.672 |
| % damage putamen | -0.111 | 0.326 | 0.456 | 726.0 | 0.485 | 0.672 |
| % damage caudate | -0.111 | 0.325 | 0.456 | 682.5 | 0.267 | 0.672 |
| % damage globus pallidus | -0.132 | 0.242 | 0.456 | 725.0 | 0.414 | 0.672 |
| Language summary z-score | -0.136 | 0.278 | 0.456 | 561.0 | 0.657 | 0.672 |
| History of prior stroke | 481.0 | 0.013* | 0.060 | 0.180 | 0.672 | 0.672 |
| Hypoperfusion | 789.5 | 0.537 | 0.683 | 0.882 | 0.348 | 0.672 |

**S7 Table.** **Relationships between demographic and stroke variables.** Spearman correlations were used to determine relationships between age and continuous or ordinal variables. Wilcoxon rank sum tests were used to determine one categorical variable and one continuous or ordinal variable. Chi-square tests were used to determine if the binary hypoperfusion variable or history of stroke varied by sex. *Stat.* = the test statistic for the corresponding tests. P-values adjusted for the False Discovery Rate (FDR) are reported as Q-values. * denotes significance at *P/Q* < 0.05, ** denotes significance at *P/Q* < 0.01.
