# Supplementary material for: The contribution of white matter pathology, hypoperfusion, lesion load, and stroke recurrence to language deficits following acute subcortical left hemisphere stroke
Source: PLoS One. 2022 Oct 26;17(10):e0275664. doi: 10.1371/journal.pone.0275664 (PMC9604977; doi:10.1371/journal.pone.0275664)
Supplement: S8 Table — Results from additional behavioral tasks are also included where relevant. (A) Patient 1 received the Western Aphasia Battery–Revised (WAB-R) and additional tests for word span, digit span, and comprehending movement-derived sentences. This patient was non-verbal, and used a communication board. (B) Patient 2 received the WAB-R, the Apraxia Battery for Adults, and additional tests for digit span, word span, and thematic role assignment. (C) Patient 3 received the Boston Diagnostic Aphasia Examination (BDAE), which included a rating scale for profiling speech characteristics. Additional tests included Pyramids & Palm Trees as well as Kissing and Dancing. (DOCX) [file pone.0275664.s008.docx]

| **A. Patient 1** |  |  |  |
| --- | --- | --- | --- |
| **Western Aphasia Battery** | | **Additional Tests** | |
| Information content | 0/10 | Digit span, forward | 5 |
| Fluency, grammatical competence | 0/10 | Digit span, backward | 0 |
| Yes/no questions | 60/60 | Word span, forward | 3 |
| Auditory word recognition | 54/60 | Word span, backward | 0 |
| Sequential commands | 40/80 | Sentence-picture matching (% correct) | 71.25 |
| Repetition | 0/100 | Enactment of spoken sentences (% correct) | 73.75 |
| Object naming | 0/60 |  |  |
| Word fluency | 0/20 |  |  |
| Sentence completion | 0/10 |  |  |
| Responsive speech | 0/10 |  |  |
| *Aphasia quotient* | *55.4/100* |  |  |
| **B. Patient 2** |  |  |  |
| **Western Aphasia Battery** | | **Apraxia Battery for Adults** | |
| Information content | 8/10 | Increasing word length, Part A (deterioration in performance) | 0/20 |
| Fluency, grammatical competence | 7/10 |  |  |
| Yes/no questions | 51/60 | Increasing word length, Part B (deterioration in performance) | 1/20 |
| Auditory word recognition | 53/60 |  |  |
| Sequential commands | 55/80 | Repeated trials | 29/30 |
| Repetition | 100/100 | Inventory of articulation (errors) | 2/15 |
| Object naming | 42/60 | **Additional Tests** | |
| Word fluency | 1/20 | Digit span, forward | 6 |
| Sentence completion | 8/10 | Digit span, backward | 5 |
| Responsive speech | 8/10 | Word span, forward | 4 |
| *Aphasia quotient* | *76.9/100* | Word span, backward | 0 |
|  |  | Thematic role assignment, video sentences | 47/80 |
|  |  | Thematic role assignment, synonym judgment (semantic condition) | 23/40 |
|  |  |  |  |
|  |  | Thematic role assignment, synonym judgment (syntactic condition) | 23/40 |
|  |  |  |  |
| **C. Patient 3** |  |  |  |
| **Boston Diagnostic Aphasia Examination** | | **Rating Scale Profile of Speech Characteristics** | |
| Simple social responses | 2/7 | Articulatory agility | 2/7 |
| Severity of speech output profile (0-5) | 1 | Phrase length | 1/7 |
| Word comprehension | 15/16 | Grammatical form | 1/7 |
| Complex ideational material | 5/6 | Sentence repetition (percentile range) | 0-20 |
| Automatized sequences | 0/4 | Auditory comprehension (percentile range) | 90-100 |
| Single word repetition | 2/5 | **Additional Tests** | |
| Sentence repetition | 0/2 | Pyramids & Palm Trees | 15/15 |
| Responsive naming | 0/10 | Kissing and Dancing | 7/15 |
| Screening of special categories | 5/12 |  |  |
| Basic symbol recognition | 4/4 |  |  |
| Number matching | 4/4 |  |  |
| Word identification, picture-word matching | 4/4 |  |  |
| Basic oral word reading (points) | 0 |  |  |
| Oral reading of sentences (# correct) | 0 |  |  |
| Well-formedness of written letters | 4/14 |  |  |
| Correctness of written letter choice | 12/21 |  |  |
| Motor facility | 4/14 |  |  |

**S8 Table. Language data from three patients with severe language deficits.** Results from additional behavioral tasks are also included where relevant. (A) Patient 1 received the Western Aphasia Battery – Revised (WAB-R) and additional tests for word span, digit span, and comprehending movement-derived sentences. This patient was non-verbal, and used a communication board. (B) Patient 2 received the WAB-R, the Apraxia Battery for Adults, and additional tests for digit span, word span, and thematic role assignment. (C) Patient 3 received the Boston Diagnostic Aphasia Examination (BDAE), which included a rating scale for profiling speech characteristics. Additional tests included Pyramids & Palm Trees as well as Kissing and Dancing.
